# Supplementary material for: Integrating Hi-C and FISH data for modeling of the 3D organization of chromosomes
Source: Nat Commun. 2019 May 3;10:2049. doi: 10.1038/s41467-019-10005-6 (PMC6499832; doi:10.1038/s41467-019-10005-6)
Supplement: Supplementary file 1 — Supplementary Information [file 41467_2019_10005_MOESM1_ESM.pdf]

Supplementary Information for:
Integrating Hi-C and FISH data for modeling of the 3D organization of chromosomes

Jianyang Zeng, et al.

1 Supplementary Notes

Supplementary Note 1

To provide more evidence of the accuracy of the 3D models reconstructed by GEM-FISH, we performed several additional validation tests. More specifically, we first compared the curves of the spatial vs. genomic distances between TADs for the models reconstructed by GEM-FISH using both Hi-C and FISH data, and GEM [1] using only Hi-C data, respectively, to those derived directly from the experimental FISH data [2]. We found that the relationships between genomic vs. inter-TAD spatial distances in the final 3D models computed by GEM-FISH were much closer to those derived from the FISH experimental data than the 3D models computed by GEM using the Hi-C data alone, especially for the regions with relatively large genomic distances (Supplementary Figures 20 - 24).

In addition, we conducted a 10-fold cross-validation procedure to further evaluate the modeling performance of our approach. In each fold, we built the average FISH distance matrix, denoted by F_{cv1}, of a specific chromosome using only 90% of the available FISH data and used it to optimize the 3D models in GEM-FISH. Then, we evaluated the reconstructed structures using the average FISH distance matrix obtained from the remaining 10% of FISH data, denoted by F_{cv2}, of that chromosome (which can be considered completely independent data). We also assessed the structures reconstructed by GEM against the same distance matrix F_{cv2}. As shown in Supplementary Table 9, GEM-FISH outperformed GEM with respect to the average relative errors against the independent distance matrix F_{cv2}.

Also, we measured the asphericity values of the 3D models (i.e., the deviation of the 3D models from being spherical) reconstructed by both GEM-FISH and GEM [3], based on the eigenvalues of their gyration tensors [4]. We observed that the models reconstructed by GEM-FISH tended to be spherical in shape, while those reconstructed by GEM tended to be more extended (Supplementary Table 10 and Supplementary Figure 25). A potential reason for these extended structures obtained by GEM is the relatively weak restraints of Hi-C contact frequencies between those TADs far away in the genomic distance.

Moreover, we inspected the radial distributions of expressed genes in the final 3D models of Chrs 20, 21, and 22 obtained by GEM-FISH and GEM, respectively (see Supplementary Note 12 for details). We observed that the expressed genes tend to lie closer to

the surface of the chromosome territories in the 3D models reconstructed by GEM-FISH than those in the 3D models reconstructed by GEM (Supplementary Figure 26), which was in agreement with the fact that active transcription regions tend to lie closer to the chromosome surface [5, 6]. We also inspected the radial distributions of the expressed genes of ChrX in the final 3D models reconstructed by GEM-FISH for both ChrXa (in which the genes are active) and ChrXi (in which the genes are inactive, except for those that escape the X-inactivation). We found that the expressed genes tend to lie closer to the chromosome surface in ChrXa than in ChrXi (Supplementary Figure 27). This result agreed well with the previous finding that the silenced genes in ChrXi tend to be shifted into a more internal location within the XIST RNA compartment [7], which provided another evidence to support the reasonableness of the final 3D models reconstructed by GEM-FISH for both ChrXa and ChrXi.

Furthermore, we used a previous finding derived from an independent FISH dataset about the spatial arrangement of genomic loci related to the nuclear pore complex (NPC) component Nup153 [8, 9], to further verify the reasonableness of our reconstructed 3D models. In particular, we first derived a number of genomic positions that are known to interact with Nup153 in Chrs 20, 21, and 22 from the DNA adenine methyltransferase identification (DamID) data [8]. We inspected the radial distribution of these genomic sites in the final 3D models of Chrs 20, 21, and 22 reconstructed by GEM-FISH and compared them to that of randomly selected loci of the same genomic lengths (denoted by Control-Nup loci) (see Supplementary Note 12 for details). We observed that most of the genomic sites interacting with Nup153 generally tend to lie closer to the surface of the chromosome territories than the Control-Nup loci (Supplementary Figure 28). Nevertheless, we also found that some sites interacting with Nup153 lie relatively close to the center of the chromosome. This observation was also consistent with the fact derived from the previous FRAP (fluorescence recovery after photobleaching) analysis that Nup153 is a nuclear basket component with a stable subpopulation at the nuclear envelope-embedded NPC, and a dynamic subpopulation exchanging between the NPC and the nucleoplasm [9]. All the above additional validation tests have further supported the superiority of our new modeling approach with independent evidences.

Supplementary Note 2

To investigate the role of Hi-C data constraints in GEM-FISH for reconstructing the TAD-level resolution models of chromosomes, we also implemented a modified version of GEM-FISH that used FISH data alone (i.e., without using the Hi-C data constraints). We ran this program ten times for each chromosome and compared the reconstructed 3D models with those obtained by GEM-FISH. We found that the average relative errors with respect to experimental FISH distances were almost the same as in GEM-FISH for all the five tested chromosomes (Supplementary Table 11). However, the Pearson correlation coefficients between the inverses of the Hi-C contact frequencies and the corresponding

spatial distances derived from the 3D models reconstructed by GEM-FISH were significantly higher than those with respect to the 3D models reconstructed using only FISH data (Supplementary Figure 29). These results demonstrated the main advantage of GEM-FISH in ensuring the consistency between the reconstructed 3D models and both Hi-C and FISH data.

Supplementary Note 3

In [10], Rao et al. observed that there are at least six nuclear subcompartments defined based on their long-range interaction patterns. They mainly used the interchromosomal interaction frequency information from Hi-C data to annotate the subcompartments. Unfortunately, due to the sparsity of the interchromosomal Hi-C maps for most of the cell lines inspected, they only annotated the subcompartments of the cell line GM12878. Recently, Di Pierro et al. [11] used the chromatin immunoprecipitation-sequencing (ChiP-Seq) data of histone marks and other protein-binding experiments to infer the subcompartment types of genomic loci. They partitioned the chromosomes into individual loci of size 50 Kbp and assigned each locus with a discrete score from 1 to 20 representing the enrichment of a certain chromatin mark. They then used a recurrent neural network that takes the scores of different chromatin marks of a certain locus and its adjacent loci as input to infer its subcompartment type. Although their model yielded high accuracy ($\sim$90%) when assigning loci to A/B compartments, it produced a large number of mismatches when assigning loci to subcompartments (A1 vs. A2, B1 vs. B2 vs. B3) [11]. Thus, in our work, although we followed the same strategy of assigning each 50 Kbp locus with a discrete score representing the enrichment of a chromatin mark as in [11], we adopted a simple way to determine the regions that carry the epigenomic content of subcompartments B1 or B2. We mainly considered the subcompartment types B1 and B2, particularly because they are expected to have different folding properties due to their distinct epigenomic content. For instance, the genomic regions belonging to subcompartment B1 with a repressive nature are expected to be more densely packed than those belonging to subcompartment B2 with an inactive nature [12].

Given that the subcompartment B2 is depleted from the marks H3K27me3, H3K36me3, H3K27ac, H3K4me1, and H3K79me2 [10], we considered the loci with a score of at most '2' (i.e., 0.1 of the maximum possible score) for all these marks to belong to subcompartment B2. Allowing the loci to have at most 0.1 of the maximum score was to account for experimental noise that may affect our analysis. On the other hand, given the relative enrichment of subcompartment B1 with the repressive mark H3K27me3, and their depletion from the activating mark H3K36me3 [10], we considered those loci with a score larger than or equal to '10' (i.e., the median score) for the mark H3K27me3, and a score of at most '2' (i.e., 0.1 of the maximum possible score) for the mark H3K36me3 to belong to subcompartment B1. We observed that the genomic loci from a certain subcompartment (B1 or B2) tend to colocalize in the 3D models reconstructed by GEM-FISH (Fig. 5(a),

5(c), and 5(e) in the main text), which was consistent with the previous finding in [10]. We also investigated the densities of regions belonging to the two subcompartments in the reconstructed 3D models and observed that the densities of the regions from subcompartment B1 were significantly higher than those of the regions from subcompartment B2 (Fig. 5(b), 5(d), and 5(f) in the main text), which was also consistent with the previous result in [12].

To verify the above simple approach that we used for identifying subcompartments B1 and B2, we have also evaluated the chromatin states of our classified regions using an independent genome annotation strategy, which was mainly adapted from [12]. More specifically, in [12], the authors defined the active regions as those enriched with the mark H3K79me3 or H3K4me2 and the repressed regions as those enriched with the mark H3K27me3. They also defined the inactive regions as those enriched with unmodified histone H3 and depleted of binding from PcG proteins and transcriptional activators. They calculated the enrichment of the mark H3K4me2 or H3K79me3 relative to H3 as $\log_2$(H3K4me2 reads/H3 reads) or $\log_2$(H3K79me3 reads/H3 reads). For the repressed domains, they calculated the enrichment of H3K27me3 as $\log_2$(H3K27me3 reads/H3 reads). For the inactive domains, they calculated the enrichment of unmodified histone H3 as (1 - $\log_2$(H3K4me2 reads/H3 reads) - $\log_2$(H3K79me3 reads/H3 reads) - $\log_2$(H3K27me3 reads/H3 reads) - $\log_2$(H3K9me2 reads/H3 reads)) [12]. For the cell line IMR90, the ChiP-Seq profiles for unmodified histone H3, H3K79me3 and H3K9me2 are not available on the ENCODE project web site. Thus, to define the active regions, we used H3K36me3 instead of H3K79me3. For the repressed regions, we used H3K27me3, and 'Control' from the ENCODE profiles as an alternative to unmodified histone H3. We calculated the average enrichment of the marks H3K27me3, H3K36me3, H3K4me2, and unmodified histone H3 for the regions that we classified (using our simple method described previously) as subcompartments B1 and B2, using the same scheme as described in [12]. We found that the regions that we classified as carrying the epigenomic content of subcompartments B1 and B2 were relatively enriched with H3K27me3 and unmodified histone H3, respectively, and both were depleted of other marks (Supplementary Table 12). This result was consistent with the repressive nature of subcompartment B1 and inactive nature of subcompartment B2, and thus supported the reasonableness of our simple scheme for classifying subcompartments B1 and B2.

Supplementary Note 4

Using their developed program HiCCUPS, Rao et al. annotated the loops in the genomes of nine different cell types from the high-resolution (1∼10 Kbps) Hi-C maps [10]. To validate these high-resolution chromatin loops of the 3D models derived from GEM-FISH, we first used the lists of the loops of Chr20, Chr21, Chr22, and ChrX of the IMR90 cell line annotated from [10] to obtain the genomic positions of the corresponding loop loci. We then calculated the spatial distance between every two loop loci in our final 3D model

computed by GEM-FISH. For each annotated loop, we also computed the spatial distance between one of the two loop anchor loci and a control locus that was located at the same genomic distance but on the opposite side of the genome. We found that the DNA packing densities of the regions between loop anchor loci were significantly higher than those of the regions between control loci for all five tested chromosomes (Supplementary Figure 14).

Supplementary Note 5

We further investigated the 3D models reconstructed by GEM-FISH for individual TADs and the organizations of histone marks within them. We first calculated the relative enrichment of the four histone marks H3K27ac, H3K4me1, H3K4me3, and H3K9me3 in individual TADs of Chrs 20, 21, and 22 using the same approach as described in [2]. More specifically, for every TAD, we calculated the read count density of each mark by dividing its read count by the genomic length of the corresponding TAD. After that, we divided the read count density of each mark in every TAD by the mean read count density of that mark along the whole chromosome to obtain its relative enrichment within the corresponding TAD. Supplementary Tables 13, 14, and 15 show the relative enrichments of the four marks mentioned above within each TAD in the three autosomes. We focused on those TADs that are highly active or inactive and investigated their structures and how different histone marks are organized within them. We considered a TAD as highly enriched with a specific mark if its relative count density is at least three times its mean count density of the whole chromosome. Accordingly, TADs that are highly enriched with one or more of the active histone marks (i.e., H3K27ac, H3K4me1, and H3K4me3) were considered highly active TADs (hereafter referred to as active TADs). Similarly, those TADs highly enriched with the mark H3K9me3 were considered highly inactive TADs (hereafter referred to as inactive TADs).

For the two types of TADs that we focused on (i.e., active and inactive TADs), it was not surprising to see that most of the loci (of 5 Kbp size) within a TAD carry the same epigenetic nature of the whole TAD (Supplementary Figure 15). On the other hand, we found that some of the loci have read count densities less than the mean read count density of the whole chromosome for all of the four marks. Moreover, we found that a small number of loci in the active TADs have inactive nature, and vice versa (Supplementary Figure 15). In addition, we noticed that the inactive TADs tend to have compact 3D models, while the 3D models of the active ones tend to be more open, which was consistent with previous findings [12] (Supplementary Figure 15). To quantitatively measure the compactness of the 3D models of both active and inactive TADs, we also calculated their mean asphericity values, which were 0.14 and 0.05 for active and inactive TADs, respectively. These results indicated that the inactive TADs tend to be more spherical, which was consistent with their compact 3D conformations, while the active TADs tend to be more extended, which was consistent with their open structures observed.

Supplementary Note 6

In principle, two adjacent TADs are allowed to overlap in the 3D models reconstructed by GEM-FISH. The overlapping fraction between two adjacent TADs depends on the locations of their centers and the shapes of their 3D structures. Following the same definition as in [12], we defined the overlapping regions between adjacent TADs as the fractions of loci in one TAD that are within 80 nm (spatial distance) from the nearest loci in the adjacent TAD. We looked into the amount of overlapping between sequential TADs along the genomic distance in Chrs 21 and 22, in which almost every pair of adjacent TADs were imaged in the original FISH data [2] that we used. We observed that adjacent TADs along the genomic distance that belong to the same compartment tend to display higher overlapping than those that belong to different compartments (Supplementary Table 2), which was consistent with the previous findings [12].

Supplementary Note 7

To test whether the tendency to lie close to the chromosome surface is specific to super-enhancers, we also compared the spatial distributions of super-enhancers vs. regular enhancers. We first obtained the list of regular enhancers in human genome from the FANTOM5 project [13,14]. Next, we compared the normalized radial distances of super-enhancers to those of regular enhancers for Chrs 20, 21, and 22 (see Supplementary Note 12 for details). As shown in Supplementary Figure 19, the analysis on our reconstructed 3D models demonstrated that super-enhancers tend to lie closer to the chromosome surface than regular enhancers.

Supplementary Note 8

Due to the cell-to-cell variability, the spatial distance between two genomic loci can change from one cell to another. The results from FISH experiments can be used to discover the distribution of the spatial distance between a pair of genomic loci. On the other hand, the Hi-C experiments capture only the interaction between two genomic loci if they are close to each other in the 3D space, which may only happen in a small population of the cells inspected. In [15], Fudenberg and Imakaev examined the Hi-C and FISH data obtained from [10] and showed that, although the median spatial distance of each loop-control pairs of loci changes concordantly with the corresponding Hi-C signal, this concordance does not always hold when comparing loop loci and control loci (i.e., loci that do not form loops) from different pairs. However, they noticed that the median spatial distance changes concordantly with the Hi-C signals for all pairs of control loci.

In the FISH data [2] used in our tests, the imaged loci span the central 100 Kbps segments of individual TADs. Each pair of these imaged loci belongs to different TADs and unlikely forms a loop. Thus, the pairs of imaged loci were basically equivalent to those control loci investigated in [15]. To examine the consistency between Hi-C and FISH

data used in our tests, we measured how often the median distances between TAD pairs measured from FISH varied concordantly with the Hi-C contact frequencies. As shown in Supplementary Table 3, the average consistency between Hi-C and FISH data for Chr20, Chr21, and Chr22 was 82.03%, which was much higher than that of the control maps. Thus, we can conclude that both Hi-C and FISH data used in our study were reasonably consistent with each other.

Supplementary Note 9

To incorporate the cell-to-cell variability of FISH data into our approach, we also proposed a new variant of GEM-FISH, called GEM-FISH*, which also took into account the cell-to-cell variability of FISH data into the modeling process. In particular, in GEM-FISH*, we assigned different weights to distinct pairwise TAD distances. The intuition was to assign higher weights to those distances with smaller variances. More specifically, in GEM-FISH*, the term corresponding to the FISH distances (C_3) is now defined as follows,

$$C_3 = \sum_i \sum_j \frac{1}{\sigma_{ij}} (||s_i - s_j|| - F_{ij})^2, \tag{1}$$

where F_{ij} and σ_{ij} denote the mean and standard deviation of spatial distances between the two TADs t_i and t_j measured from FISH experiments, respectively, and the symbols s_i and s_j stand for the coordinates of the centers of TADs t_i and t_j, respectively.

We ran GEM-FISH* 100 times on the datasets of Chrs 20, 21, 22, Xa, and Xi, and compared the results with those obtained using GEM-FISH. As shown in Supplementary Table 16, the modeling results from GEM-FISH and GEM-FISH* were quite close to each other. Thus, we sticked to using the original version of GEM-FISH.

Supplementary Note 10

The FISH data of the IMR90 cell line obtained from [2] did not provide any direct information about the pairwise distances between genomic loci inside individual TADs. Nonetheless, we can still derive rough estimates of the volume, size, and radius of gyration R_g of every TAD using the available FISH data, which can still provide useful information for modeling the 3D structures of individual TADs.

The FISH data derived from [2] of a specific chromosome, denoted by Chr_i, include a set of n structures, where n stands for the number of imaged copies of Chr_i. Each structure of Chr_i consists of m three-dimensional points, each corresponding to a TAD probed in that chromosome. The genomic regions in Chr_i probed by FISH experiments in [2] either cover all its TADs (as in Chr21 and Chr22) or span approximately uniform intervals in the chromosome (as in Chr20 and ChrX).

For each of the n structures of Chr_i, based on the FISH data measured in [2], we first derive an estimate of its volume (i.e., the volume of the minimum bounding box (mbb)

circumscribing that structure) and its size (i.e., the length of the longest side of the mbb), and also calculate its radius of gyration, denoted by R_g. We then plot histograms to show the distributions of volumes and sizes for individual structures of Chr_i and select the values corresponding to the highest values to be the expected volume and size of that chromosome. We also infer an approximate linear relation between the size of the chromosome and its radius of gyration (Supplementary Figures 30 - 34).

We use V_{chr_i} and S_{chr_i} to denote the expected volume and size of Chr_i, respectively. Given that the volume of a genomic region basically scales linearly with its genomic length [16], a rough estimate of the volume of TAD_j in Chr_i can be derived by:

$$V_{tad_j} = V_{chr_i} \times \frac{L_{tad_j}}{L_{chr_i}}, \tag{2}$$

where L_{tad_j} and L_{chr_i} stand for the genomic lengths of TAD_j and Chr_i, respectively.

Since the ratio between the sizes of TAD_j and Chr_i (which are denoted by S_{tad_j} and S_{chr_i}, respectively) is approximately equal to the third power of the corresponding ratio of their volumes, we have

$$S_{tad_j} = S_{chr_i} \times (\frac{V_{tad_j}}{V_{chr_i}})^{\frac{1}{3}}. \tag{3}$$

Using the approximate linear relation that we previously inferred between the size of the chromosome and its radius of gyration (Supplementary Figures 30 - 34), we can obtain an estimate of the radius of gyration ($\hat{R_g}$) of TAD_j. After that, we can place this estimated value into Eq. 7 in the main text and use it as an additional constraint to model the 3D structure of TAD_j.

We have also inspected the modeling performance of GEM-FISH when using the mean and median values of the volumes and sizes of individual chromosomes to derive the approximate values of R_g. We found that the average relative errors with respect to experimental FISH data for the final 3D models reconstructed by GEM-FISH were close to each other, when using the mode (i.e., the value corresponding to the highest peak in the distribution), mean, and median values to estimate the values of R_g for individual TADs (Supplementary Table 17).

Supplementary Note 11

In our work, we applied grid search to find the best pair of parameter values that yield high scores of the scoring function (see Eq. 18) mainly using the data from Chr21 and Chr22. We found that the values $\lambda_E = 5 \times 10^{12}$ and $\lambda_F = 10^{-8}$ led to the highest score for Chr22 and a reasonably high score for Chr21. We further investigated how the parameter settings can change the value of the score function and affect the reconstructed 3D model. Supplementary Figure 35(a) shows the values of the scoring function when fixing $\lambda_E = 5 \times 10^{12}$ and changing λ_F from 1×10^{-6} to 1×10^{-10}. Similarly, Supplementary

Figure 35(b) shows the values of the scoring function when fixing $\lambda_F = 1 \times 10^{-8}$ and changing λ_E from 5×10^{10} to 5×10^{14}.

To examine how different parameter settings may affect the reconstructed models, we ran GEM-FISH ten times with different combinations of parameters λ_E and λ_F. Supplementary Figure 36 shows the mean values of average relative errors obtained for Chrs 20, 21, and 22. Supplementary Figures 37 and 38 show the Pearson correlation coefficients between pairwise spatial distances between TADs derived from the 3D models reconstructed by GEM-FISH vs. inverse Hi-C contact frequencies and experimental FISH distances, respectively. We also calculated the harmonic means of Pearson correlation coefficients with both FISH distances and inverse Hi-C contact frequencies to measure the agreement between the reconstructed models with both Hi-C and FISH data (Supplementary Figure 39). All these tests demonstrated that the values of our chosen parameters $\lambda_F = 1 \times 10^{-8}$ and $\lambda_E = 5 \times 10^{12}$ produced reasonably good modeling results.

To further test the robustness of the choice of parameters λ_E and λ_F on other Hi-C datasets, we also repeated all the above experiments using Hi-C data from [17] with resolution 40 Kbp. Supplementary Figures 40 - 43 show that the combination of parameters $\lambda_F = 1 \times 10^{-8}$ and $\lambda_E = 5 \times 10^{12}$ still yielded good results even on this different Hi-C dataset. Thus, most likely our choice of the parameters is optimal and robust for Hi-C data at a range of resolution. In principle, the method we provided (see Section 4.5) or a cross validation like procedure can be easily used to determine the optimal parameter setting for any input data.

Supplementary Note 12

To obtain the normalized radial distance of a certain point P corresponding to a specific locus in our reconstructed 3D models, we first calculated the centroid of the 3D model, denoted as C. We then obtained R, the Euclidean distance of the farthest point in the 3D model from the centroid. After that, we divided the Euclidean distance between the two points P and C by the largest distance R to obtain the normalized radial distance of point P.

2 Supplementary Discussion

We evaluated how the dynamics of the chromosome conformations experimentally measured by FISH experiments in [2] deviated from the average 3D models reconstructed by GEM-FISH. We first calculated the volumes of the minimum bounding boxes that enclosed the spatial positions of TADs experimentally measured in [2] for individual copies of the five chromosomes. We then grouped the copies of each chromosome measured in the FISH experiments into ten groups according to their volumes and calculated the average distance matrix for each group. After that, we compared the derived average distance

matrix of each group with the average 3D model reconstructed by GEM-FISH. We found that in general the more the number of structures that each group contained, the less the relative error obtained when comparing the average 3D model reconstructed by GEM-FISH with the average distance matrix of that group (Supplementary Figures 44 - 48). In addition, we plotted the average 3D model reconstructed by GEM-FISH for each of the five chromosomes and compared it visually with the experimentally obtained FISH conformations of five randomly picked imaged copies. This visual comparison showed that, due to the dynamic nature of chromosomes, their conformations may display a certain level of deviation from each other and also from the average 3D model reconstructed by GEM-FISH (Supplementary Figure 49).

In addition, we examined if the incorporation of the cell-to-cell variability in the FISH data can enhance the performance of GEM-FISH. In particular, we implemented a new variant of GEM-FISH, called GEM-FISH*, which also took into account the cell-to-cell variability of FISH data by assigning different weights to distinct pairwise TAD distances (more details can be found in Supplementary Note 9). We observed that the modeling results from GEM-FISH and GEM-FISH* were quite close to each other (Supplementary Table 16). Thus, we sticked to using the original version of GEM-FISH.

3 Supplementary Tables

Supplementary Table 1. Comparison between different modeling approaches, including GEM-FISH, MDS, Shrec3D, chromosome3D, and ChromSDE in terms of average relative errors and the number of TADs correctly assigned to the two different compartments. For Shrec3D, chromosome3D, and ChromSDE, due to the availability of only the non-allele-specific Hi-C maps (which do not distinguish between ChrXa and ChrXi) for the IMR90 cell line [10], we calculated only one 3D model for ChrX, and compared the assignment of its TADs to the two different compartments against those obtained by experimental FISH data for Chrs Xa and Xi, respectively. To avoid unfair comparison, we did not calculate the average relative errors with respect to experimental FISH distances for the 3D models derived by Shrec3D, chromosome3D, and ChromSDE.

	Average relative error		Number of TADs correctly assigned					Asphericity				
	GEM-FISH	MDS	GEM-FISH	chromosome3D	MDS	Shrec3D	chromSDE	GEM-FISH	chromosome3D	MDS	Shrec3D	chromSDE
Chr20	0.178	0.356	26.89	17	16	19	25	0.025	0.24	0.089	0.245	0.288
Chr21	0.162	0.312	31.92	28.4	32	17	29	0.024	0.66	0.055	0.106	0.413
Chr22	0.175	0.355	21.86	18.6	24	18	23	0.027	0.13	0.072	0.218	0.113
ChrXa	0.173	0.362	38.96	38.4	40	38	39	0.07	0.23	0.244	0.591	0.762
ChrXi	0.222	0.522	36.19	21.4	33	22	21	0.005	0.23	0.069	0.591	0.762

Supplementary Table 2. Average overlapping fractions between adjacent TADs along the genomic distance belonging to the same compartment (i.e., with the same epigenetic nature), and those belonging to different compartments (i.e., with different epigenetic nature). Source data are provided as a Source Data file.

	Average overlapping fraction	
	TADs belonging to the same compartment	TADs belonging to different compartments
Chr21	0.15	0.06
Chr22	0.06	0.02

Supplementary Table 3. Consistency between the median spatial distances between the TAD pairs from FISH data [2] and their corresponding Hi-C contact frequencies [10] used in our tests, which was defined as the percentage of median spatial distances that vary concordantly with the corresponding Hi-C contact frequencies. The control maps were randomly generated with non-negative numbers, satisfying the symmetric property and having the same sizes as the Hi-C maps.

	Hi-C maps [10]	Control maps
Chr20	79.67%	48.72%
Chr21	83.79%	49.13%
Chr22	82.63%	49.78%
Average	82.63%	49.21%

Supplementary Table 4. Mean and standard deviation values of average relative errors and asphericity values for the 3D models reconstructed by running GEM-FISH and GEM 100 times. Source data are provided as a Source Data file.

	Average relative error (for 100 runs)		Asphericity (for 100 runs)	
	GEM-FISH	GEM	GEM-FISH	GEM
Chr20	0.178 ± 0.002	0.317 ± 0.009	0.025 ± 0.003	0.187 ± 0.009
Chr21	0.162 ± 0.002	0.326 ± 0.032	0.024 ± 0.002	0.308 ± 0.018
Chr22	0.175 ± 0.003	0.223 ± 0.009	0.027 ± 0.003	0.103 ± 0.005
ChrXa	0.173 ± 0.001	1.341 ± 0.022	0.072 ± 0.002	0.256 ± 0.053
ChrXi	0.222 ± 0.001	1.981 ± 0.033	0.005 ± 0.001	0.256 ± 0.053

Supplementary Table 5. Mean and standard deviation values of the numbers of correctly assigned TADs to the two different compartments in the 3D models generated by running GEM-FISH and GEM 100 times. Source data are provided as a Source Data file.

		Number of TADs correctly assigned to the two different compartments (for 100 runs)	
	Number of TADs	GEM-FISH	GEM
Chr20	30	26.89 ± 0.72	22.21 ± 0.41
Chr21	34	31.92 ± 0.46	29.99 ± 0.10
Chr22	27	21.86 ± 1.25	20.51 ± 0.68
ChrXa	40	38.96 ± 0.71	35.86 ± 1.15
ChrXi	40	36.20 ± 1.48	23.36 ± 0.89

Supplementary Table 6. Mean and standard deviation values of average relative errors and the numbers of TADs correctly assigned to the two different compartments for an ensemble of four 3D models at TAD-level resolution computed by GEM-FISH (using the same optimization technique as in [1]) and GEM. In total, 100 trials were repeated. Source data are provided as a Source Data file.

		Average relative error (for 100 runs)		Number of TADs correctly assigned to the two different compartments (for 100 runs)	
	Number of TADs	GEM-FISH (ensemble)	GEM (ensemble)	GEM-FISH (ensemble)	GEM (ensemble)
Chr20	30	0.176 ± 0.003	0.283 ± 0.021	26.89 ± 0.65	23.22 ± 1.05
Chr21	34	0.159 ± 0.002	0.262 ± 0.023	32.80 ± 0.67	28.50 ± 0.51
Chr22	27	0.175 ± 0.002	0.221 ± 0.112	22.12 ± 1.32	21.96 ± 0.60
ChrXa	40	0.163 ± 0.007	1.192 ± 0.019	38.94 ± 0.57	39.45 ± 0.74
ChrXi	40	0.203 ± 0.009	1.783 ± 0.032	36.39± 1.32	20.93 ± 0.81

Supplementary Table 7. Time needed by GEM-FISH to reconstruct the 3D models of Chrs 20, 21, 22, Xa, and Xi at the TAD-level resolution on a server with 32 2.40GHz Intel(R) Xeon(R) CPUs and 128GB RAM.

	Number of TADs	Time
Chr20	30	55.4 sec
Chr21	34	91.1 sec
Chr22	27	82.5 sec
ChrXa	40	54.1 sec
ChrXi	40	67.1 sec

Supplementary Table 8. Time needed by GEM-FISH to reconstruct the 3D models of the most time-demanding intra-TAD models for Chrs 20, 21, 22, Xa, and Xi at 5 Kbp, 10 Kbp, 25 Kbp, and 50 Kbp resolutions, respectively. The computations were carried on a server with 32 2.40GHz Intel(R) Xeon(R) CPUs and 128GB RAM.

			5 Kbp resolution		10 Kbp resolution		25 Kbp resolution		50 Kbp resolution	
	TAD ID	TAD size	Number of loci	Running time	Number of loci	Running time	Number of loci	Running time	Number of loci	Running time
Chr20	TAD 19	1.72 Mbp	344	0.46 hr	172	5.72 min	69	0.75 min	35	0.48 min
Chr21	TAD 2	2.88 Mbp	576	7.96 hr	288	77.41 min	116	4.86 min	58	1.67 min
Chr22	TAD 6	3.12 Mbp	624	1.03 hr	312	9.28 min	125	2.46 min	63	1.32 min
ChrXa	TAD 25	7.88 Mbp	1576	21.02 hr	788	69 min	316	7.41 min	158	2.76 min
ChrXi	TAD 25	7.88 Mbp	1576	25.21 hr	788	89.45 min	316	7.74 min	158	2.46 min

Supplementary Table 9. Mean and standard deviation values of average relative errors when testing the 3D models reconstructed by GEM-FISH (using Hi-C data and only 90% of the available FISH data) and GEM (using only Hi-C data) against the remaining 10% of the available FISH data. In GEM, due to the availability of only the non-allele-specific Hi-C maps (which did not distinguish between ChrXa and ChrXi) for the IMR90 cell line [10], we only considered one 3D model for the X chromosome, and computed the average relative errors with respect to experimental FISH distances for ChrXa and ChrXi, respectively. Source data are provided as a Source Data file.

	GEM-FISH	GEM
Chr20	0.210 ± 0.014	0.352 ± 0.037
Chr21	0.188 ± 0.010	0.362 ± 0.026
Chr22	0.205 ± 0.011	0.237 ± 0.007
ChrXa	0.217 ± 0.021	1.407 ± 0.096
ChrXi	0.258 ± 0.016	1.995 ± 0.115

Supplementary Table 10. Mean and standard deviation of asphericity values for the 3D models reconstructed by GEM-FISH and GEM for Chrs 20, 21, and 22. Source data are provided as a Source Data file.

	GEM-FISH	GEM
Chr20	0.026 ± 0.003	0.187 ± 0.009
Chr21	0.025 ± 0.001	0.308 ± 0.018
Chr22	0.027 ± 0.002	0.102 ± 0.005

Supplementary Table 11. Mean and standard deviations of average relative errors, number of TADs correctly assigned to the two different compartments, and asphericity values for the 3D models obtained by GEM-FISH and a modified version of GEM-FISH that uses only FISH data. Source data are provided as a Source Data file.

	Average relative error (for 10 runs)		Number of TADs correctly assigned to the two different compartments (for 10 runs)		Asphericity (for 10 runs)	
	GEM-FISH	FISH only	GEM-FISH	FISH only	GEM-FISH	FISH only
Chr20	0.177 ± 0.002	0.177 ± 0.001	27.0 ± 0.47	25.9 ± 1.59	0.023 ± 0.003	0.016 ± 0.003
Chr21	0.162 ± 0.002	0.162 ± 0.002	32.0 ± 0.0	31.5 ± 1.17	0.024 ± 0.001	0.023 ± 0.002
Chr22	0.175 ± 0.003	0.175 ± 0.001	22.0 ± 1.33	22.8 ± 1.13	0.026 ± 0.002	0.021 ± 0.005
ChrXa	0.173 ± 0.001	0.175 ± 0.002	39.0 ± 0.66	36.8 ± 1.47	0.068 ± 0.002	0.06 ± 0.006
ChrXi	0.222 ± 0.001	0.220 ± 0.001	36.5 ± 0.97	35.0 ± 3.36	0.005 ± 0.001	0.003 ± 0.001

Supplementary Table 12. Average relative enrichment of active and repressive marks, and unmodified histone H3 in the regions belonging to B1 and B2 subcompartments, according to the annotation method adopted from [12]. The regions belonging to subcompartment B1 are enriched with repressive marks (shown in bold) and depleted from other marks. On the other hand, the regions belonging to subcompartment B2 are enriched with unmodified histone H3 (shown in bold) and depleted from other marks. Source data are provided as a Source Data file.

	Average relative enrichment for H3K36me3			Average relative enrichment for H3K4me2			Average relative enrichment for H3K27me3			Average relative enrichment for unmodified histones H3		
	Chr20	Chr21	Chr22	Chr20	Chr21	Chr22	Chr20	Chr21	Chr22	Chr20	Chr21	Chr22
B1	-0.65	-0.808	-0.585	-0.48	-0.389	-0.22	**2.607**	**2.412**	**2.646**	-0.47	-0.214	-0.839
B2	-0.588	-0.731	-0.308	-1.152	-0.852	-0.751	-1.099	-0.751	-1.048	**3.836**	**3.336**	**2.699**

Supplementary Table 13. Relative enrichments of the four histone marks H3K27ac, H3K4me3, H3K4me1, and H3K9me3 in individual TADs of Chr20. The first column gives the TAD IDs. Starting from the second column, the relative enrichments of different histone marks are shown. The last column shows the compartment type (A or B) to which the corresponding TAD belongs based on the experimental FISH data.

TAD ID	H3K27ac	H3K4me3	H3K4me1	H3K9me3	Compartment type
1	1.2533	1.0769	2.3423	0.1792	B
2	1.0997	1.3349	1.9609	0.044422	A
3	1.5672	1.7151	1.2254	0.10597	A
4	0.25724	0.28628	0.16611	1.9484	B
5	1.3515	1.5201	0.4258	0.1678	B
6	1.4085	1.4533	0.75819	0.13018	A
7	0.21047	0.26546	0.18646	2.4291	B
8	2.3675	2.0946	2.0705	0.10707	A
9	3.678	2.7348	2.6779	0.1628	B
10	0.2554	0.29176	0.39154	2.3938	B
11	0.28823	0.46292	0.061763	0.36297	B
12	0.92925	1.0228	1.5356	0.27981	B
13	1.4302	1.4559	2.3535	0	A
14	2.5531	1.9316	1.914	0	A
15	2.1441	1.8826	2.8375	0.030241	A
16	1.1463	1.4809	2.9747	0.067632	A
17	1.983	1.9689	2.4053	0.08052	A
18	0.27708	0.38227	0.37573	1.036	B
19	0.043236	0.058956	0.041513	3.6944	B
20	1.4638	1.5144	1.5367	0.020581	B
21	1.7681	1.9125	0.74647	0.2581	A
22	0.64117	0.81788	0.67933	0.61837	B
23	4.6047	3.7281	2.357	0.014197	A
24	1.5525	2.4961	0.57214	0.082788	A
25	0.32346	0.34631	0.29235	1.6904	B
26	0.34689	0.48343	0.27621	1.3976	B
27	1.3685	0.76515	0.79919	0.75668	B
28	0.83384	1.0979	1.3842	0.94582	B
29	1.4894	1.9201	4.3024	0.64394	B
30	0.86723	1.2888	1.8641	0.25342	A

Supplementary Table 14. Relative enrichments of the four histone marks H3K27ac, H3K4me3, H3K4me1, and H3K9me3 in individual TADs of Chr21. The first column gives the TAD IDs. Starting from the second column, the relative enrichments of different histone marks are shown. The last column shows the compartment type (A or B) to which the corresponding TAD belongs based on the experimental FISH data.

TAD ID	H3K27ac	H3K4me3	H3K4me1	H3K9me3	Compartment type
1	0.066121	0.11234	0.11188	0	B
2	0.35199	0.3326	0.42734	0.7882	B
3	0.42806	0.45713	0.45325	4.6223	B
4	0.035074	0.037609	0.029822	0	B
5	0.22153	0.2023	0.10736	3.4503	B
6	0.22973	0.24349	0.061523	1.9897	B
7	0.71911	0.73595	0.65694	1.1378	B
8	1.7157	1.1834	1.5848	0.096008	A
9	2.5705	1.3378	0.77653	0.078021	A
10	3.9996	2.6775	2.6235	0.14027	B
11	0.18502	0.22066	0.057273	2.0196	B
12	0.43046	0.7076	0.69793	0.1196	B
13	1.003	1.2654	1.5061	0.038453	B
14	1.608	1.5698	2.7392	0.044627	B
15	0.81681	0.97178	2.2287	0.072702	B
16	1.6472	1.7203	2.3571	0.066515	A
17	1.1825	1.5195	1.3719	0.22553	A
18	1.8373	1.8173	1.9218	0.12838	A
19	1.9488	2.0044	1.329	0.091923	A
20	1.6917	1.7521	2.4646	0	A
21	1.3239	1.6015	1.6348	0.061662	A
22	1.9747	1.7593	2.8412	0.14462	A
23	0.83026	1.228	0.14867	0.17169	B
24	2.1349	1.9168	1.9892	0.087209	B
25	0.068909	0.10956	0.0705	2.2964	B
26	0.61415	1.1013	0.7304	0.086245	B
27	1.6713	2.0781	1.7716	0.067741	B
28	0.46854	1.0518	0.49014	0.17288	B
29	2.0593	2.3235	3.0439	0.064511	A
30	1.2481	1.7221	2.1419	0.025235	A
31	0.57811	0.91977	1.3115	0.82738	A
32	3.4785	4.2077	4.6165	0.17265	A
33	2.5004	3.1646	1.3252	0.17626	A
34	3.0405	2.1949	3.7568	0.17125	A

Supplementary Table 15. Relative enrichments of the four histone marks H3K27ac, H3K4me3, H3K4me1, and H3K9me3 in individual TADs of Chr22. The first column gives the TAD IDs. Starting from the second column, the relative enrichments of different histone marks are shown. The last column shows the compartment type (A or B) to which the corresponding TAD belongs based on the experimental FISH data.

TAD ID	H3K27ac	H3K4me3	H3K4me1	H3K9me3	Compartment type
1	0.11987	0.14318	0.33149	0.019873	B
2	1.644	1.5354	1.3694	0	B
3	0.75268	0.87985	1.5888	0.19873	A
4	2.0136	2.1152	2.0241	0.059932	A
5	1.1341	0.98852	1.5944	0.01929	A
6	0.77154	0.97012	0.86268	0.14898	B
7	0.25244	0.34606	0.28414	2.2569	B
8	1.1984	1.2885	1.5038	0.10302	B
9	0.77795	1.0254	0.26341	0.21236	B
10	1.1169	1.1106	0.95507	0.10159	B
11	2.0458	1.5014	1.1554	0.15699	A
12	1.4218	1.2623	1.7387	0.078958	A
13	1.2035	1.0784	0.99836	0.10466	B
14	0.29694	0.37824	0.30716	3.4149	B
15	1.8809	1.758	0.91581	0.060577	A
16	7.4318	4.3269	2.1933	0.066531	A
17	0.34163	0.48826	0.77141	0.1093	A
18	1.3156	1.9216	0.61794	0.14545	A
19	1.7049	1.4703	2.3014	0.050093	A
20	1.0276	1.1462	1.3895	0.13844	A
21	1.8558	1.3486	2.0468	0.020299	A
22	0.65483	0.79087	1.329	0.16252	A
23	2.0579	2.1285	1.7475	0.053529	A
24	1.0103	1.2647	0.61599	0.35916	B
25	1.4445	1.4849	1.3342	0.24128	A
26	0.14466	0.23419	0.060766	5.4926	B
27	1.3022	1.319	2.4121	0.12267	A

Supplementary Table 16. Mean and standard deviation values of average relative errors, numbers of correctly assigned TADs to the two different compartments, and asphericity values for the 3D models generated by GEM-FISH and GEM-FISH* (which also took into account the cell-to-cell variability of FISH data), respectively. Source data are provided as a Source Data file.

	Average relative error (for 100 runs)		Number of TADs correctly assigned to the two different compartments (for 100 runs)		Asphericity (for 100 runs)	
	GEM-FISH	GEM-FISH*	GEM-FISH	GEM-FISH*	GEM-FISH	GEM-FISH*
Chr20	0.178 ± 0.002	0.183 ± 0.002	26.89 ± 0.72	27.09 ± 0.933	0.025 ± 0.003	0.034 ± 0.005
Chr21	0.162 ± 0.002	0.164 ± 0.002	31.92 ± 0.47	32.41 ± 0.933	0.024 ± 0.002	0.030 ± 0.004
Chr22	0.175 ± 0.003	0.176 ± 0.002	21.86 ± 1.26	21.69 ± 0.872	0.027 ± 0.003	0.033 ± 0.003
ChrXa	0.173 ± 0.001	0.175 ± 0.001	38.96 ± 0.71	38.01 ± 1.04	0.071 ± 0.002	0.071 ± 0.003
ChrXi	0.222 ± 0.001	0.224 ± 0.001	36.19 ± 1.48	35.67 ± 1.74	0.005 ± 0.001	0.007 ± 0.001

Supplementary Table 17. Average relative errors with respect to experimental FISH data for the final 3D models reconstructed by GEM-FISH for Chrs 20, 21, 22, Xa, and Xi, when using the highest, median, and mean values of the volumes of the chromosomes and their sizes to estimate the values of the radius of gyration of individual TADs, respectively.

	Using highest value	Using median value	Using mean value
Chr20	0.163	0.163	0.162
Chr21	0.143	0.139	0.137
Chr22	0.159	0.157	0.157
ChrXa	0.157	0.157	0.157
ChrXi	0.211	0.211	0.210

4 Supplementary Figures

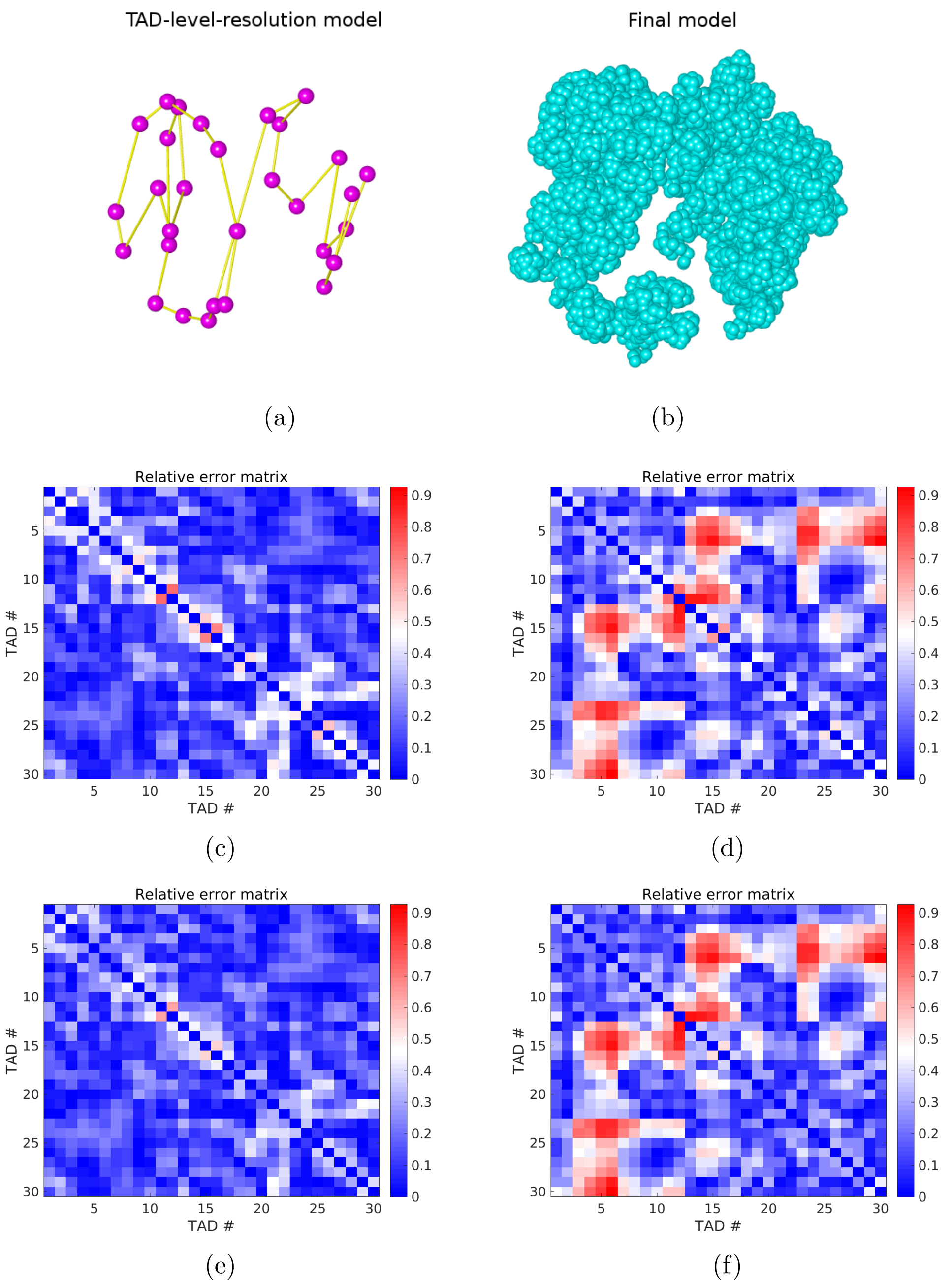

Supplementary Figure 1. The modeling results of human Chromosome 20 (Chr20). (a) The TAD-level resolution 3D structure of Chr20 calculated by GEM-FISH, where each dot represents the center of a TAD. (b) The final 3D structure of Chr20 reconstructed by GEM-FISH. The visualization in (a) and (b) was performed using UCSF Chimera [18]. (c and d) The relative error matrices of the TAD-level resolution models computed by GEM-FISH using both Hi-C and FISH data, and by GEM using only Hi-C data, respectively. (e and f) The relative error matrices of the final models computed by GEM-FISH using both Hi-C and FISH data, and GEM using only Hi-C data, respectively.

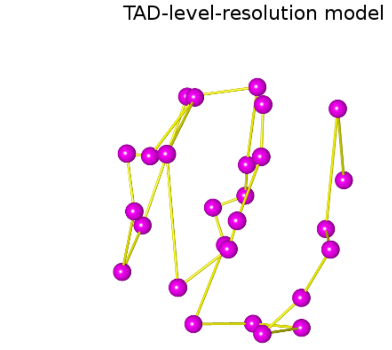
TAD-level-resolution model

(a)

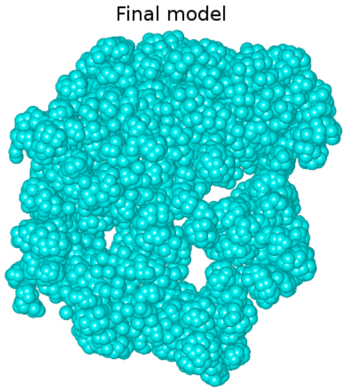
Final model

(b)

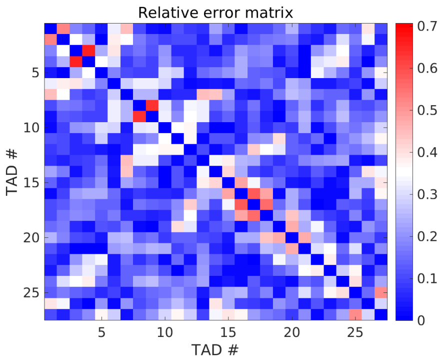
Relative error matrix
TAD #
TAD #
0.7
0.6
0.5
0.4
0.3
0.2
0.1
0

(c)

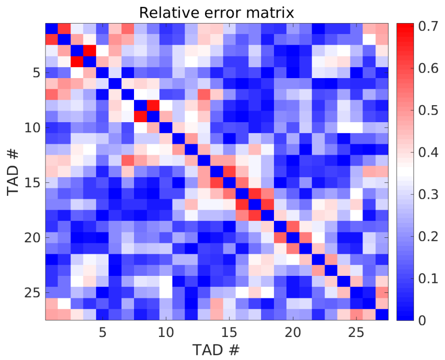
Relative error matrix
TAD #
TAD #
0.7
0.6
0.5
0.4
0.3
0.2
0.1
0

(d)

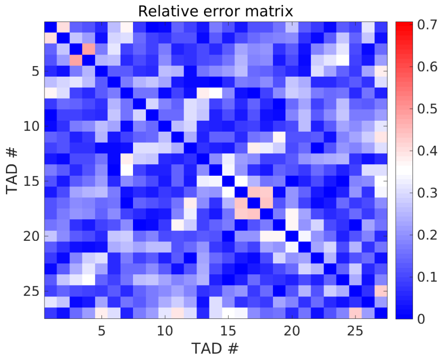
Relative error matrix
TAD #
TAD #
0.7
0.6
0.5
0.4
0.3
0.2
0.1
0

(e)

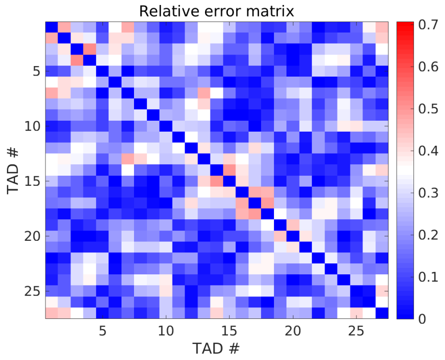
Relative error matrix
TAD #
TAD #
0.7
0.6
0.5
0.4
0.3
0.2
0.1
0

(f)

Supplementary Figure 2. The modeling results of human Chromosome 22 (Chr22). (a) The TAD-level resolution 3D structure of Chr22 calculated by GEM-FISH, where each dot represents the center of a TAD. (b) The final 3D structure of Chr22 reconstructed by GEM-FISH. The visualization in (a) and (b) was performed using UCSF Chimera [18]. (c and d) The relative error matrices of the TAD-level resolution models computed by GEM-FISH using both Hi-C and FISH data, and by GEM using only Hi-C data, respectively. (e and f) The relative error matrices of the final models computed by GEM-FISH using both Hi-C and FISH data, and GEM using only Hi-C data, respectively.

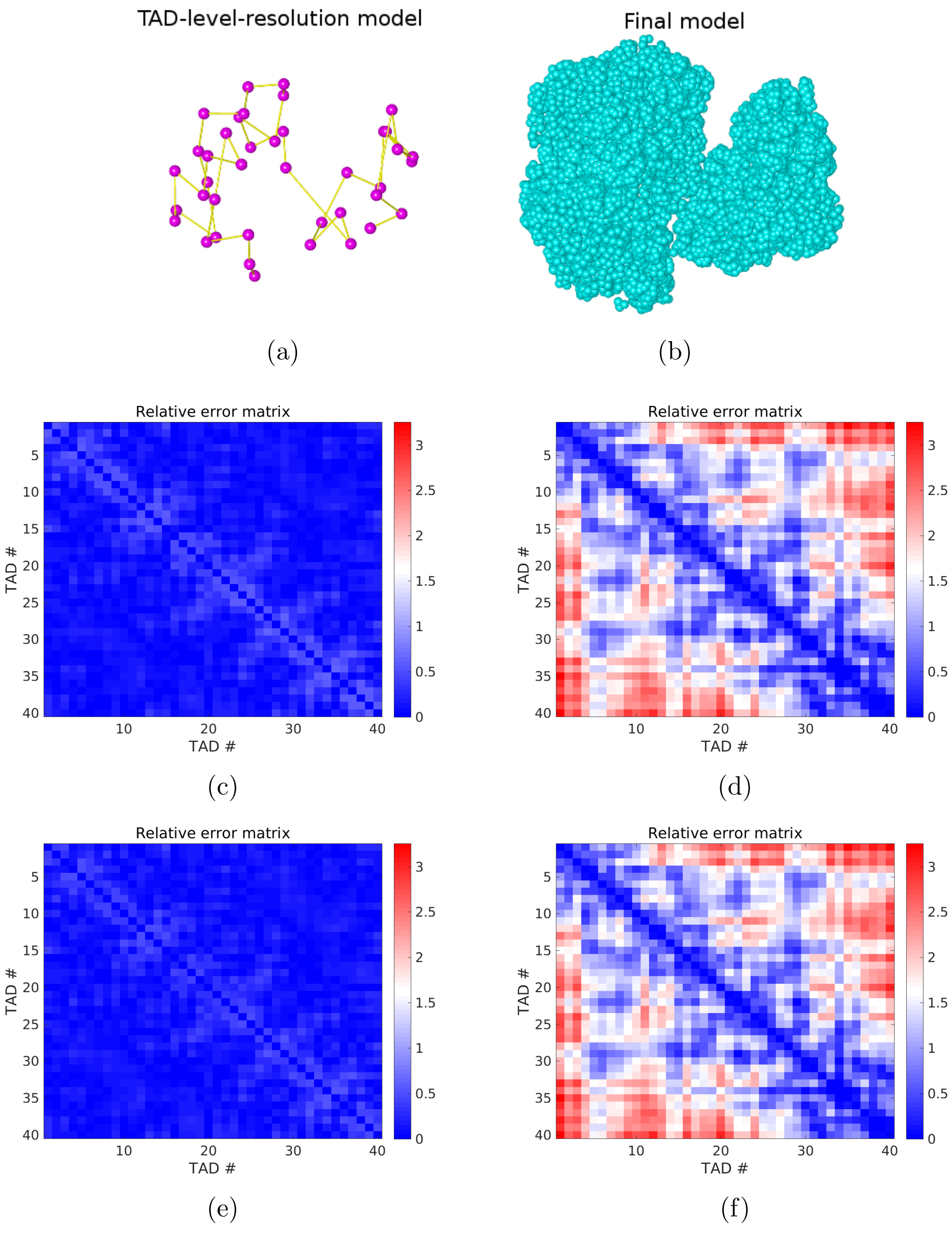
TAD-level-resolution model
Final model
(a)
(b)
Relative error matrix
TAD #
(c)
(d)
(e)
(f)

Supplementary Figure 3. The modeling results of human Chromosome Xa (ChrXa). (a) The TAD-level resolution 3D structure of ChrXa calculated by GEM-FISH, where each dot represents the center of a TAD. (b) The final 3D structure of ChrXa reconstructed by GEM-FISH. The visualization in (a) and (b) was performed using UCSF Chimera [18]. (c and d) The relative error matrices of the TAD-level resolution models computed by GEM-FISH using both Hi-C and FISH data, and by GEM using only Hi-C data, respectively. (e and f) The relative error matrices of the final models computed by GEM-FISH using both Hi-C and FISH data, and GEM using only Hi-C data, respectively.

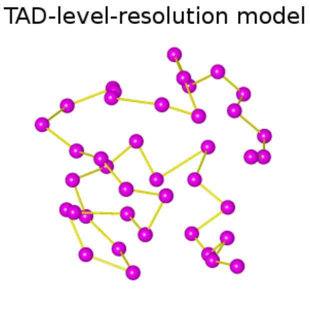
TAD-level-resolution model

(a)

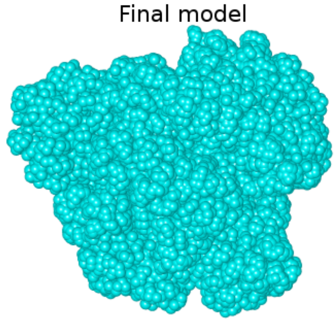
Final model

(b)

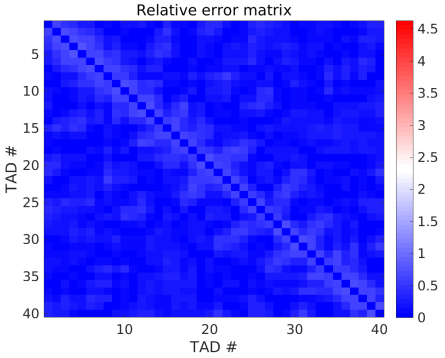
Relative error matrix
TAD #
TAD #
5
10
15
20
25
30
35
40
10
20
30
40
4.5
4
3.5
3
2.5
2
1.5
1
0.5
0

(c)

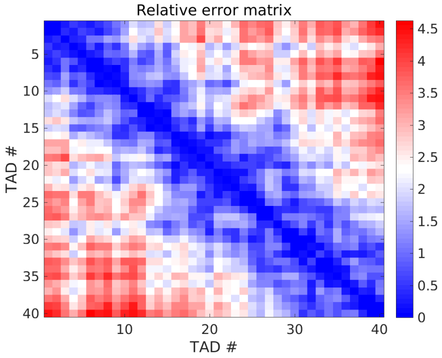
Relative error matrix
TAD #
TAD #
5
10
15
20
25
30
35
40
10
20
30
40
4.5
4
3.5
3
2.5
2
1.5
1
0.5
0

(d)

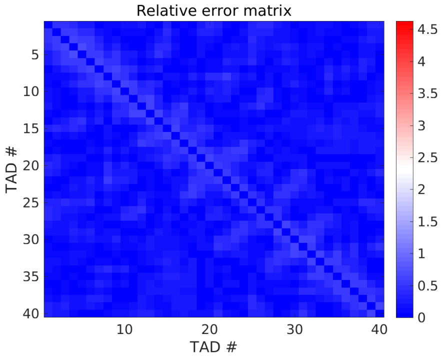
Relative error matrix
TAD #
TAD #
5
10
15
20
25
30
35
40
10
20
30
40
4.5
4
3.5
3
2.5
2
1.5
1
0.5
0

(e)

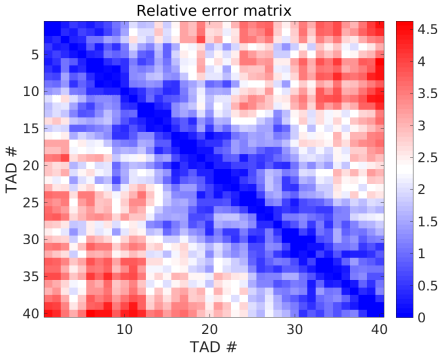
Relative error matrix
TAD #
TAD #
5
10
15
20
25
30
35
40
10
20
30
40
4.5
4
3.5
3
2.5
2
1.5
1
0.5
0

(f)

Supplementary Figure 4. The modeling results of human Chromosome Xi (ChrXi). (a) The TAD-level resolution 3D structure of ChrXi calculated by GEM-FISH, where each dot represents the center of a TAD. (b) The final 3D structure of ChrXi reconstructed by GEM-FISH. The visualization in (a) and (b) was performed using UCSF Chimera [18]. (c and d) The relative error matrices of the TAD-level resolution models computed by GEM-FISH using both Hi-C and FISH data, and by GEM using only Hi-C data, respectively. (e and f) The relative error matrices of the final models computed by GEM-FISH using both Hi-C and FISH data, and GEM using only Hi-C data, respectively.

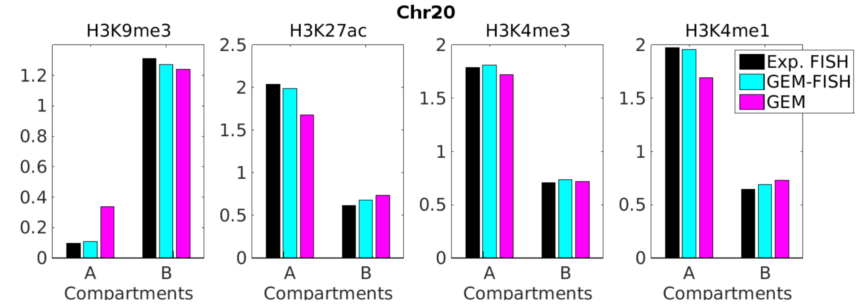

(a)

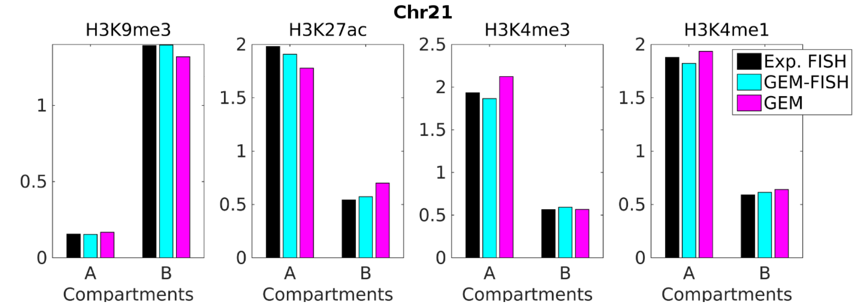

(b)

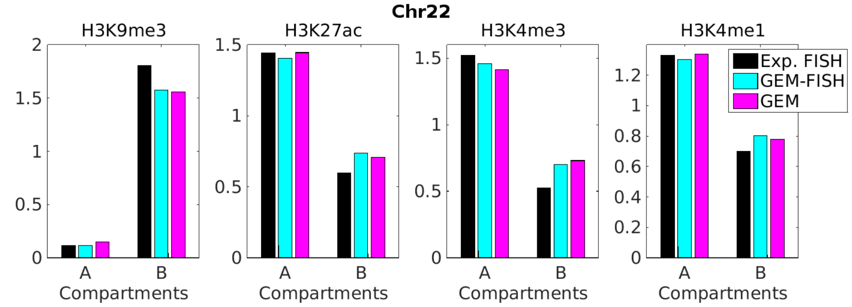

(c)

Supplementary Figure 5. Relative enrichment of different histone marks, including H3K9me3, H3K27ac, H3K4me3, and H3K4me1, on compartments A and B of Chr20 (a), Chr21 (b), and Chr22 (c) for different schemes of assigning TADs to A/B compartments, including using the experimental FISH data and the 3D chromosome models reconstructed by GEM-FISH (using both Hi-C and FISH data) and GEM (using only Hi-C data).

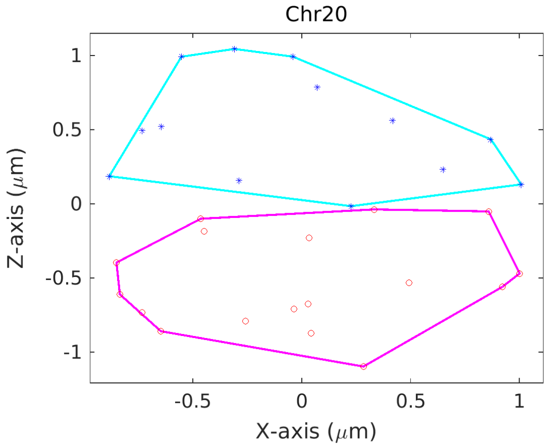
Chr20
Z-axis (μm)
X-axis (μm)
1
0.5
0
-0.5
-1
-0.5
0
0.5
1

(a)

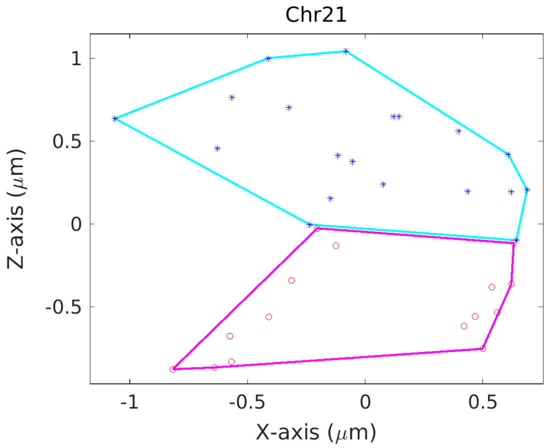
Chr21
Z-axis (μm)
X-axis (μm)
1
0.5
0
-0.5
-1
-0.5
0
0.5

(b)

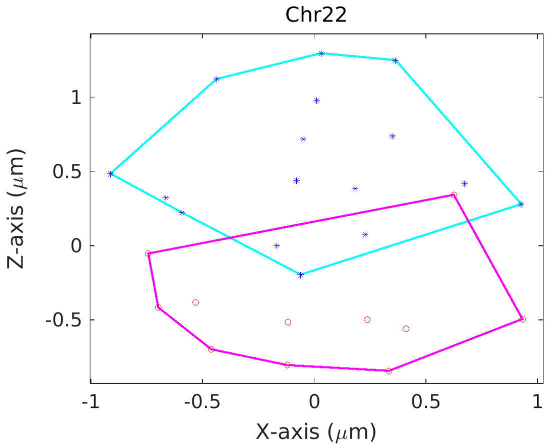
Chr22
Z-axis (μm)
X-axis (μm)
1
0.5
0
-0.5
-1
-0.5
0
0.5
1

(c)

Supplementary Figure 6. Projection of the 3D convex hull plots of the A and B compartments to the XZ plane for Chr20 (a), Chr21 (b), and Chr22 (c).

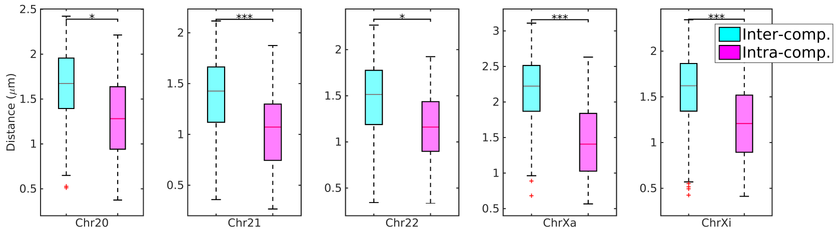

Supplementary Figure 7. Comparisons of the pairwise distances between TADs within the same compartment vs. those between TADs from different compartments. N_{inter} = 442, 570, 364, 768, and 792 pairwise distances between TADs from different compartments for Chr20, Chr21, Chr22, ChrXa, and ChrXi, respectively. N_{intra} = 428, 552, 338, 792, and 768 pairwise distances between TADs from the same compartment for Chr20, Chr21, Chr22, ChrXa, and ChrXi, respectively. *: p-value $< 10^{-26}$, **: p-value $< 10^{-46}$, ***: p-value $< 10^{-122}$. For the boxplots, the top and bottom lines of each box represent the 75th and 25th percentiles of the samples, respectively. The line inside each box represents the median of the samples. The upper and lower lines above and below the boxes are the whiskers. Red points marked by '+' represent outliers, which represent the observations beyond 1.5 times interquartile range away from the top or the bottom of the box.

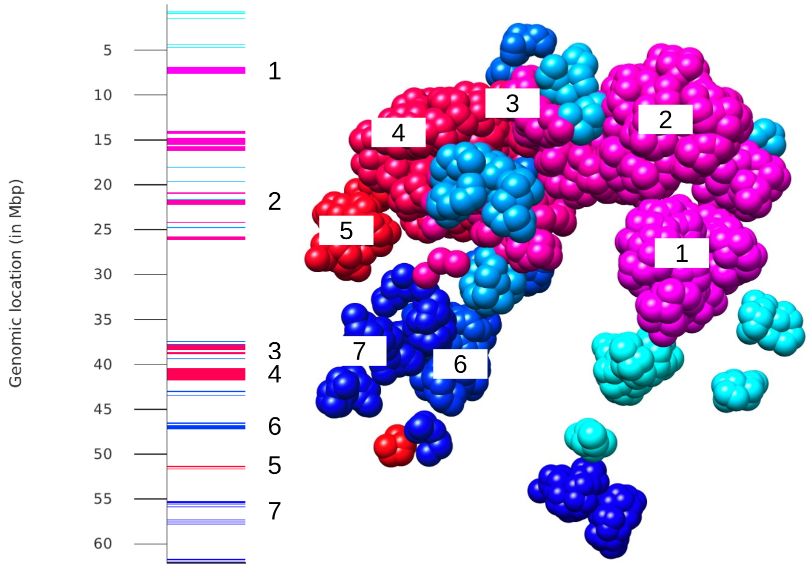

Supplementary Figure 8. Visualization of the regions belonging to subcompartments B1 and B2 in Chr20. Along the genomic distance, subcompartment B1 is represented by colors ranging from cyan to blue, while subcompartment B2 is represented by colors ranging from magenta to red. The colors become darker with the increase in genomic distance. Although the regions '1', '2', '3', '4', and '5' are far away in the genomic distance, they tend to colocalize in the 3D model. Similarly, the regions '6' and '7' also tend also to colocate in the 3D space in spite of the large genomic distance between them (∼10 Mbp). The visualization was performed mainly using UCSF Chimera [18].

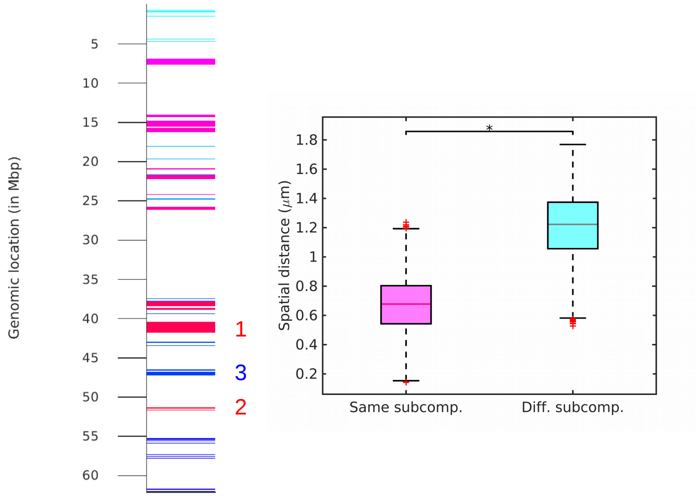
Genomic location (in Mbp)
5
10
15
20
25
30
35
40
45
50
55
60
1
3
2
Spatial distance (μm)
0.2
0.4
0.6
0.8
1
1.2
1.4
1.6
1.8
*
Same subcomp.
Diff. subcomp.

(a)

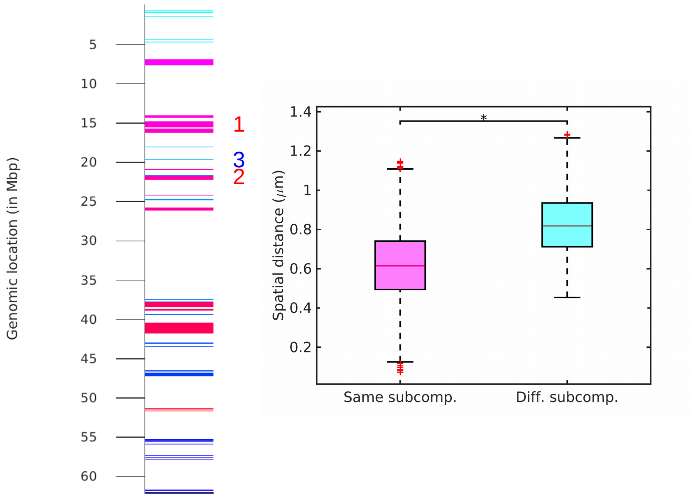
Genomic location (in Mbp)
5
10
15
20
25
30
35
40
45
50
55
60
1
3
2
Spatial distance (μm)
0.2
0.4
0.6
0.8
1
1.2
1.4
*
Same subcomp.
Diff. subcomp.

(b)

Supplementary Figure 9. Comparison of the colocalization between intra- and inter-subcompartment genomic loci for Chr20. (a and b) Spatial distances between loci in genomic regions '1' and '2' (which belong to subcompartment B2) are smaller than those between loci in genomic regions '1' and '3' (which belong to subcompartments B2 and B1, respectively), although regions '1' and '3' are closer to each other than regions '1' and '2'. $N_{same} = 15000$ and 13770 pairwise distances between points corresponding to 5 Kbp genomic loci in the genomic regions '1' and '2' for (a) and (b), respectively. $N_{diff} = 45000$ and 2700 pairwise distances between points corresponding to 5 Kbp genomic loci in the genomic regions '1' and '3' for (a) and (b), respectively. *: negligible p-value, one-tailed Wilcoxon rank sum test. For the boxplots, the top and bottom lines of each box represent the 75th and 25th percentiles of the samples, respectively. The line inside each box represents the median of the samples. The upper and lower lines above and below the boxes are the whiskers. Red points marked by '+' represent outliers, which represent the observations beyond 1.5 times interquartile range away from the top or the bottom of the box.

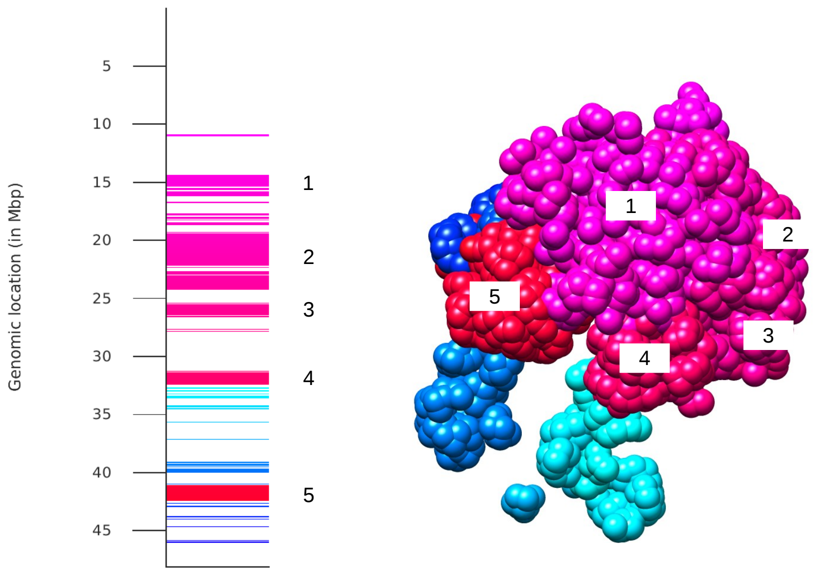

Supplementary Figure 10. Visualization of the regions belonging to subcompartments B1 and B2 in Chr21. Along the genomic distance, subcompartment B1 is represented by colors ranging from cyan to blue, while subcompartment B2 is represented by colors ranging from magenta to red. The colors become darker with the increase in genomic distance. The regions '1', '2', '3', '4', and '5' tend to colocalize in the 3D model in spite of being far away in the genomic distance. The visualization was performed mainly using UCSF Chimera [18].

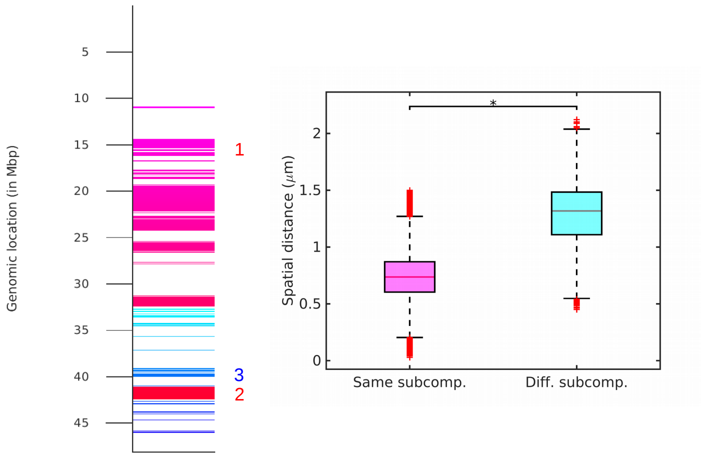
Genomic location (in Mbp)
5
10
15
20
25
30
35
40
45
1
3
2
Spatial distance (μm)
0
0.5
1
1.5
2
*
Same subcomp.
Diff. subcomp.

(a)

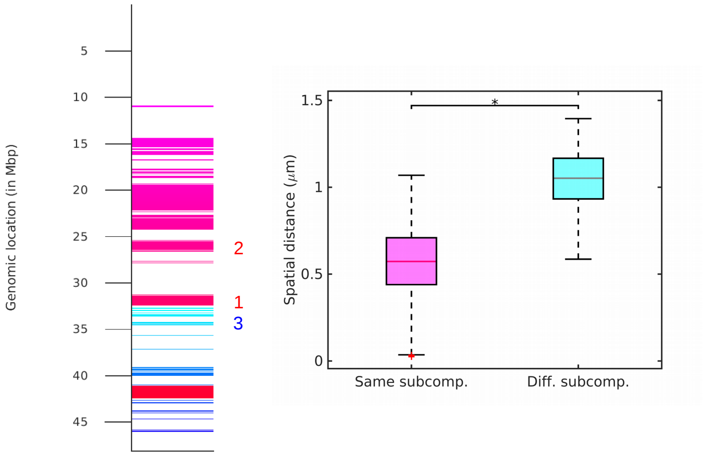
Genomic location (in Mbp)
5
10
15
20
25
30
35
40
45
2
1
3
Spatial distance (μm)
0
0.5
1
1.5
*
Same subcomp.
Diff. subcomp.

(b)

Supplementary Figure 11. Comparison of the colocalization between intra- and inter-subcompartment genomic loci for Chr21. (a and b) Spatial distances between loci in genomic regions '1' and '2' (which belong to subcompartment B2) are smaller than those between loci in genomic regions '1' and '3' (which belong to subcompartments B2 and B1, respectively), although regions '1' and '3' are closer to each other than regions '1' and '2'. N_{same} = 97720 and 59508 pairwise distances between points corresponding to 5 Kbp genomic loci in the genomic regions '1' and '2' for (a) and (b), respectively. N_{diff} = 47467 and 11400 pairwise distances between points corresponding to 5 Kbp genomic loci in the genomic regions '1' and '3' for (a) and (b), respectively. *: negligible p-value, one-tailed Wilcoxon rank sum test. For the boxplots, the top and bottom lines of each box represent the 75th and 25th percentiles of the samples, respectively. The line inside each box represents the median of the samples. The upper and lower lines above and below the boxes are the whiskers. Red points marked by '+' represent outliers, which represent the observations beyond 1.5 times interquartile range away from the top or the bottom of the box.

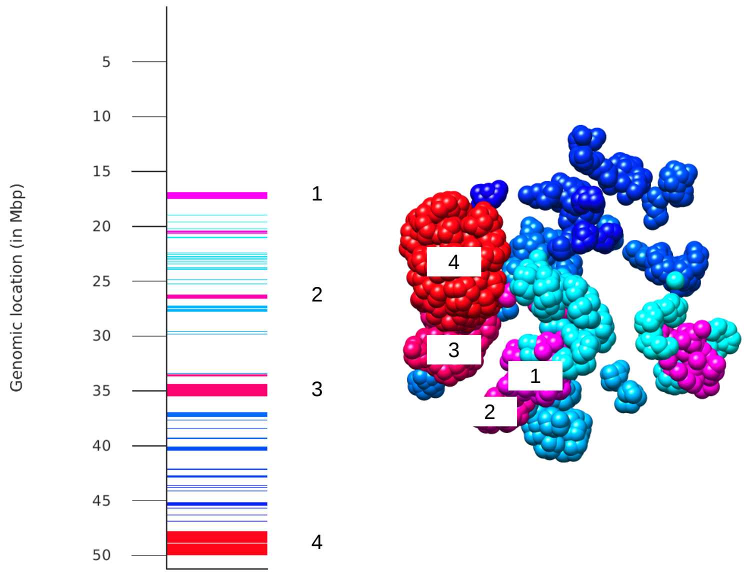

Supplementary Figure 12. Visualization of the regions belonging to subcompartments B1 and B2 in Chr22. Along the genomic distance, subcompartment B1 is represented by colors ranging from cyan to blue, while subcompartment B2 is represented by colors ranging from magenta to red. The colors become darker with the increase in genomic distance. The regions '1', and '2', and the regions '3', and '4' tend to colocalize in the 3D model in spite of being far away in the genomic distance. The visualization was performed mainly using UCSF Chimera [18].

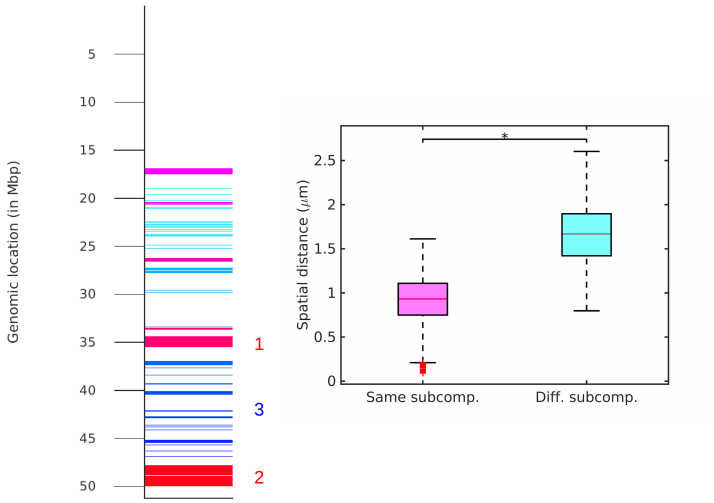
Genomic location (in Mbp)
5
10
15
20
25
30
35
40
45
50
1
3
2
Spatial distance (μm)
0
0.5
1
1.5
2
2.5
*
Same subcomp.
Diff. subcomp.

(a)

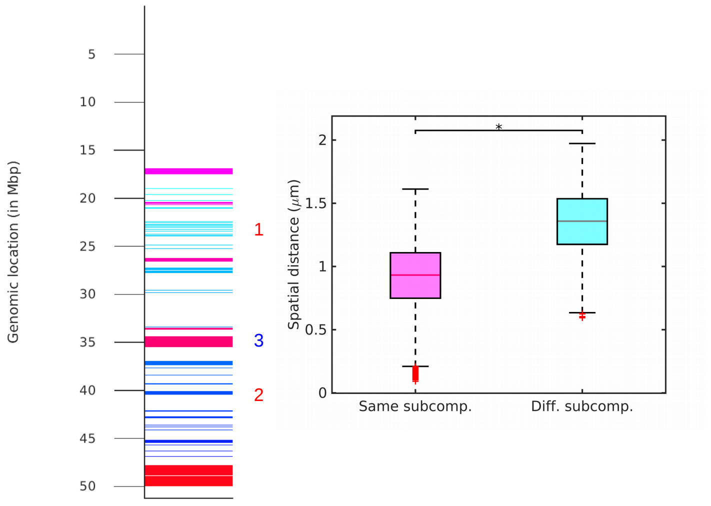
Genomic location (in Mbp)
5
10
15
20
25
30
35
40
45
50
1
3
2
Spatial distance (μm)
0
0.5
1
1.5
2
*
Same subcomp.
Diff. subcomp.

(b)

Supplementary Figure 13. Comparison of the colocalization between intra- and inter-subcompartment genomic loci for Chr22. (a and b) Spatial distances between loci in genomic regions '1' and '2' (which belong to subcompartment B2) are smaller than those between loci in genomic regions '1' and '3' (which belong to subcompartments B2 and B1, respectively), although regions '1' and '3' are closer to each other than regions '1' and '2'. $N_{same} = 111800$ and 111801 pairwise distances between points corresponding to 5 Kbp genomic loci in the genomic regions '1' and '2' for (a) and (b), respectively. $N_{diff} = 83200$ and 36800 pairwise distances between points corresponding to 5 Kbp genomic loci in the genomic regions '1' and '3' for (a) and (b), respectively. *: negligible p-value, one-tailed Wilcoxon rank sum test. For the boxplots, the top and bottom lines of each box represent the 75th and 25th percentiles of the samples, respectively. The line inside each box represents the median of the samples. The upper and lower lines above and below the boxes are the whiskers. Red points marked by '+' represent outliers, which represent the observations beyond 1.5 times interquartile range away from the top or the bottom of the box.

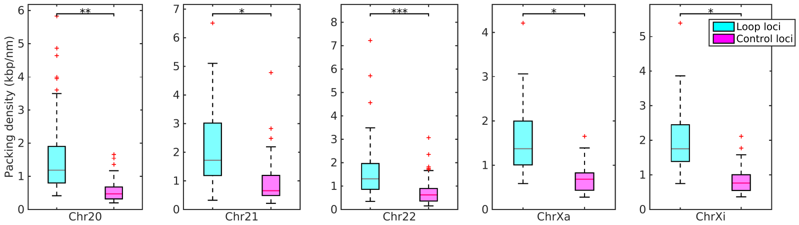

Supplementary Figure 14. Comparisons of the packing densities between loop loci vs. between control loci. $N = 92$, 48, 110, 32, and 32 genomic loci for both loop and control regions for Chr20, Chr21, Chr22, ChrXa, and ChrXi, respectively. *: p-value $< 10^{-4}$, **: p-value $< 10^{-8}$, ***: p-value $< 10^{-11}$. All tests were performed using the one-tailed Wilcoxon rank-sum test. For the boxplots, the top and bottom lines of each box represent the 75th and 25th percentiles of the samples, respectively. The line inside each box represents the median of the samples. The upper and lower lines above and below the boxes are the whiskers. Red points marked by '+' represent outliers, which represent the observations beyond 1.5 times interquartile range away from the top or the bottom of the box.

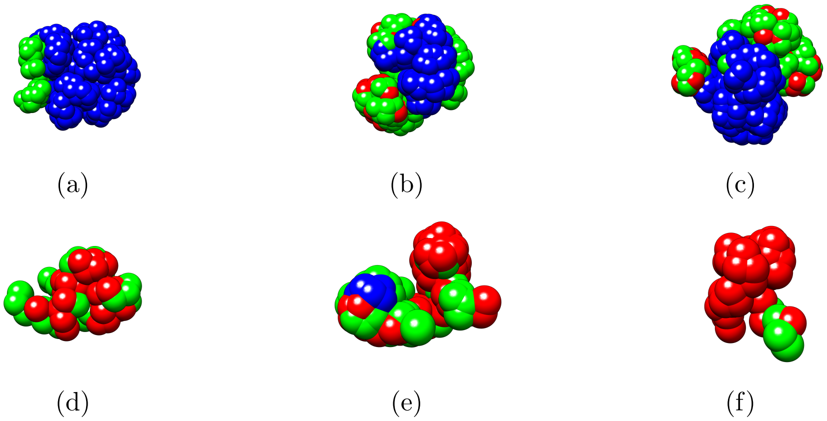

Supplementary Figure 15. Examples of the 3D models of inactive (a, b, and c) and active TADs (d, e, and f). Blue and red colors denote inactive and active loci (of 5 Kbp size), respectively. Green color denotes the loci whose read count densities for all the four marks (i.e., H3K27ac, H3K4me1, H3K4me3, and H3K9me3) are less than the mean read count density of the whole chromosome.

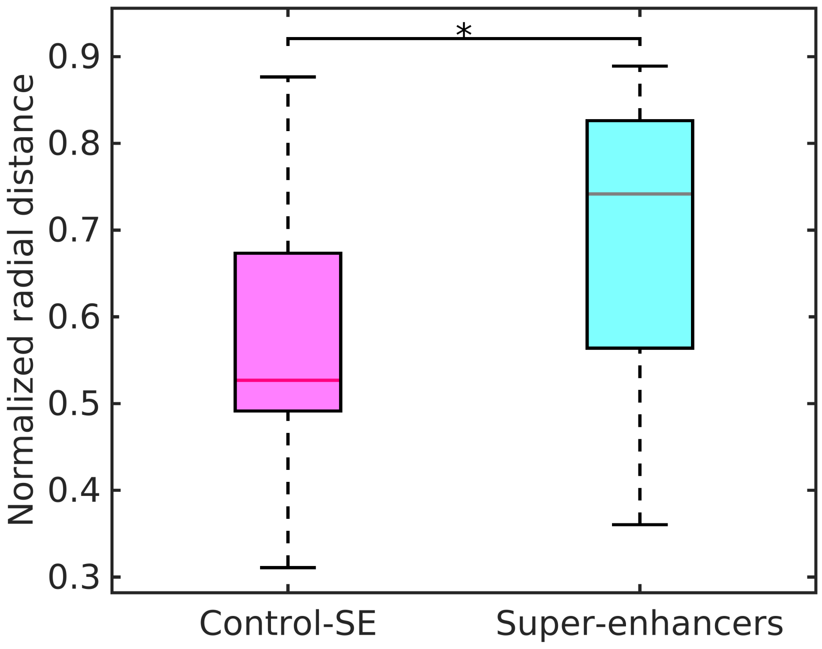

Supplementary Figure 16. Super-enhancers tend to lie closer to the chromosome surface than randomly selected control loci (denoted as “Control-SE”) of the same genomic lengths . The modeling results of Chrs 20, 21, and 22 were used in the analysis. $N = 84$ points in the final 3D models, corresponding to the 5 Kbp genomic loci in Chrs 20, 21, and 22 for both super-enhancers and control loci. *: p-value $< 10^{-5}$, one-tailed Wilcoxon rank sum test. For the boxplots, the top and bottom lines of each box represent the 75th and 25th percentiles of the samples, respectively. The line inside each box represents the median of the samples. The upper and lower lines above and below the boxes are the whiskers. Red points marked by ‘+’ represent outliers, which represent the observations beyond 1.5 times interquartile range away from the top or the bottom of the box.

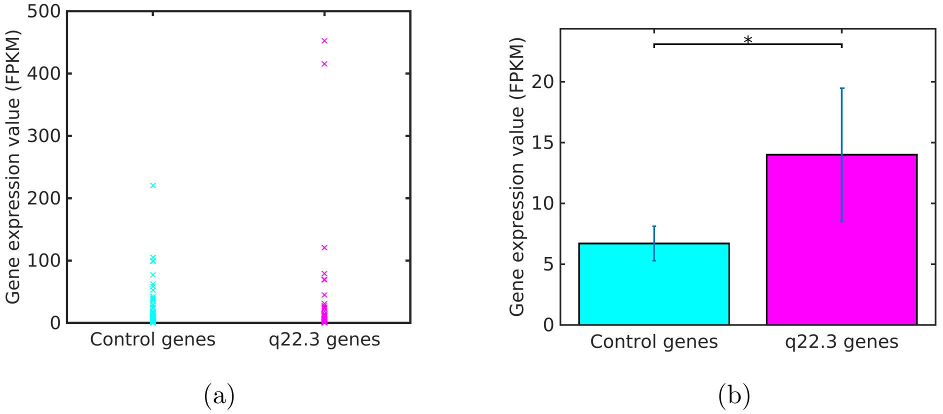

Supplementary Figure 17. Expression values of the genes in the region q22.3 of Chr21 are significantly higher than those in the other regions of the same chromosome (referred to as control genes). (a) Dot plots representing the gene expression values of the genes in the region q22.3 of Chr21 and other conteol genes. (b) A bar graph in which the two bars represent the mean expression values for the genes in the region q22.3 and the control genes, respectively. The error bars represent the standard errors of the two samples. $N = 225$ and 115 genes for the control set and the region q22.3, respectively. *: p-value = 0.048, one-tailed two-sample t-test. Source data are provided as a Source Data file.

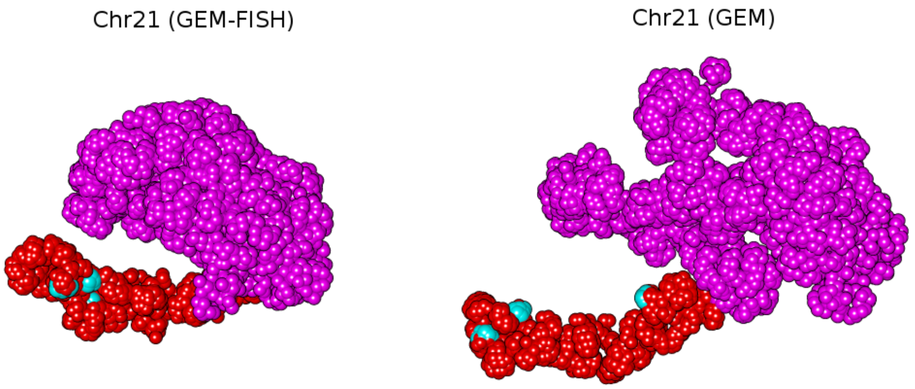

Supplementary Figure 18. Comparison between the 3D models of Chr21 reconstructed by GEM-FISH (left) using both Hi-C and FISH data and GEM (right) using Hi-C data alone. The visualization was performed using UCSF Chimera [18]. In both 3D models, the gene-rich G-band q22.3 is shown in red, while the remaining parts are shown in magenta. The super-enhancers are shown in cyan.

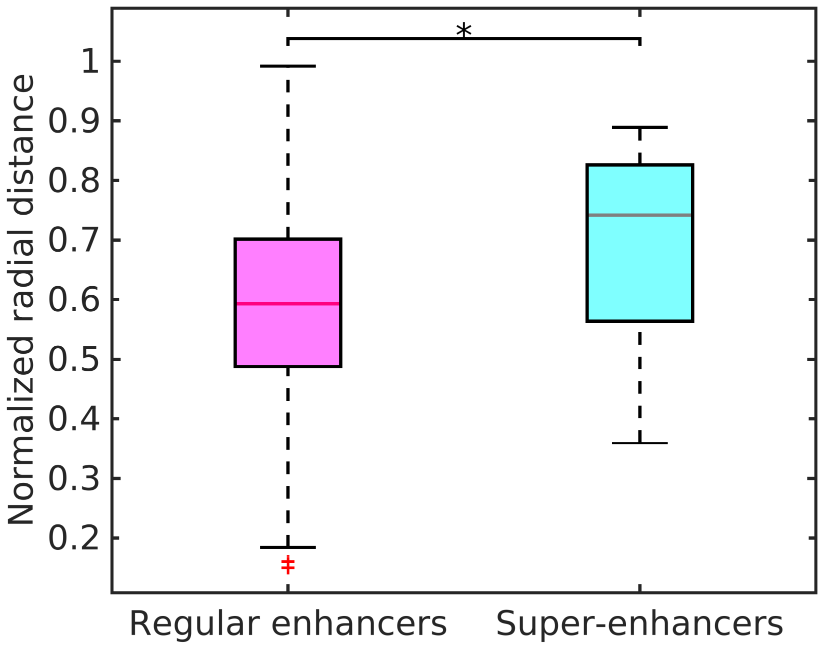

Supplementary Figure 19. Super-enhancers tend to lie closer to the chromosome surface than regular enhancers. The modeling results of Chrs 20, 21, and 22 were used in the analysis. *: p-value $< 10^{-7}$, one-tailed Wilcoxon rank sum test. $N_{se} = 84$ points in the final 3D models, corresponding to the 5 Kbp genomic loci of super-enhancers in Chrs 20, 21, and 22. $N_{re} = 2347$ points in the final 3D models, corresponding to the 5 Kbp genomic loci of regular enhancers in Chrs 20, 21, and 22. For the boxplots, the top and bottom lines of each box represent the 75th and 25th percentiles of the samples, respectively. The line inside each box represents the median of the samples. The upper and lower lines above and below the boxes are the whiskers. Red points marked by '+' represent outliers, which represent the observations beyond 1.5 times interquartile range away from the top or the bottom of the box.

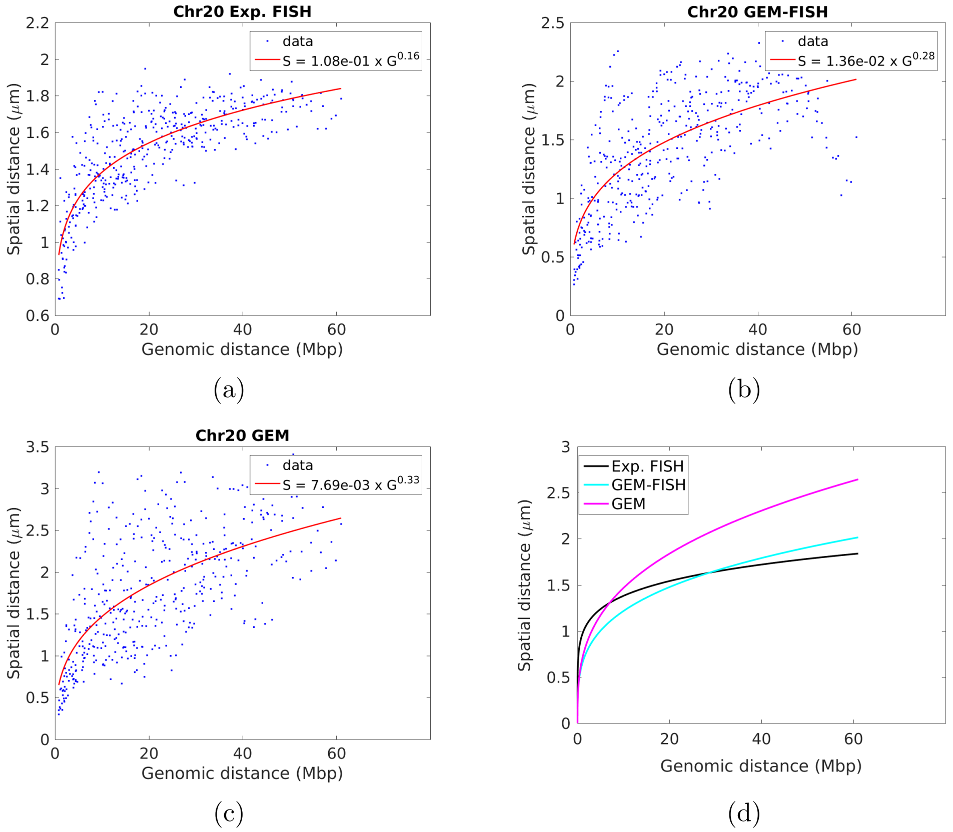

Supplementary Figure 20. The scatter plots and the corresponding fitting curves of spatial vs. genomic distances between TADs for Chr20, which were derived from the experimental FISH data (a), and the final 3D models reconstructed by GEM-FISH (b), and by GEM (c). (d) A combined figure for the three fitting curves shown in (a, b, and c).

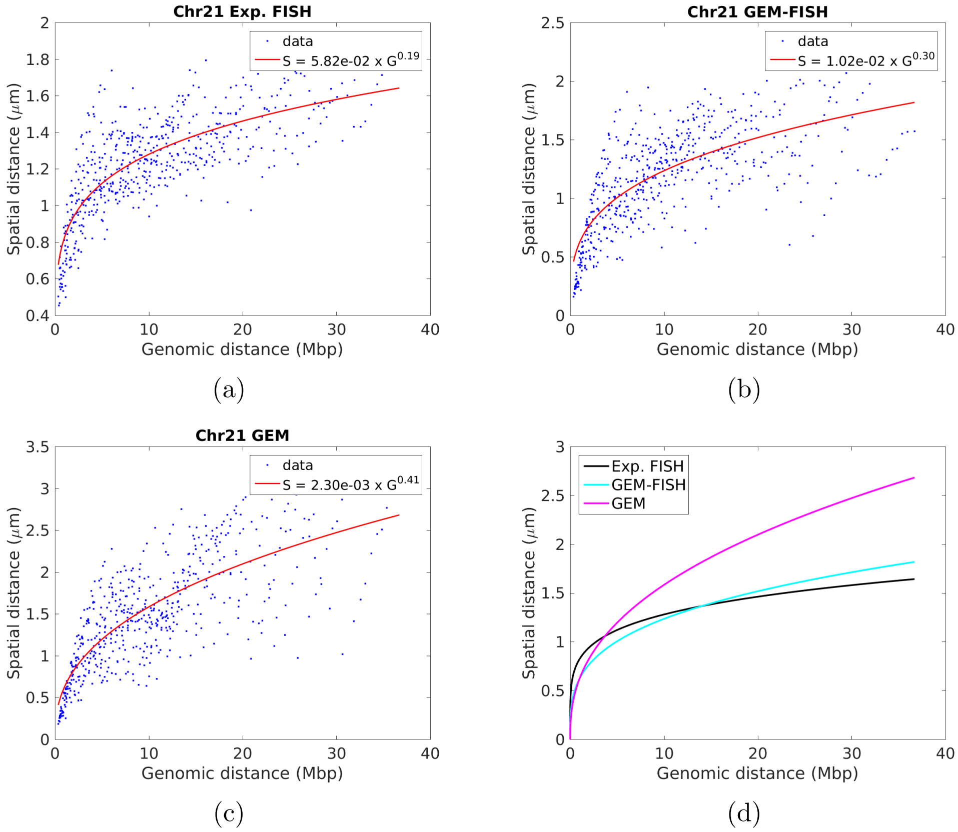

Supplementary Figure 21. The scatter plots and the corresponding fitting curves of spatial vs. genomic distances between TADs for Chr21, which were derived from the experimental FISH data (a), and the final 3D models reconstructed by GEM-FISH (b), and by GEM (c). (d) A combined figure for the three fitting curves shown in (a, b, and c).

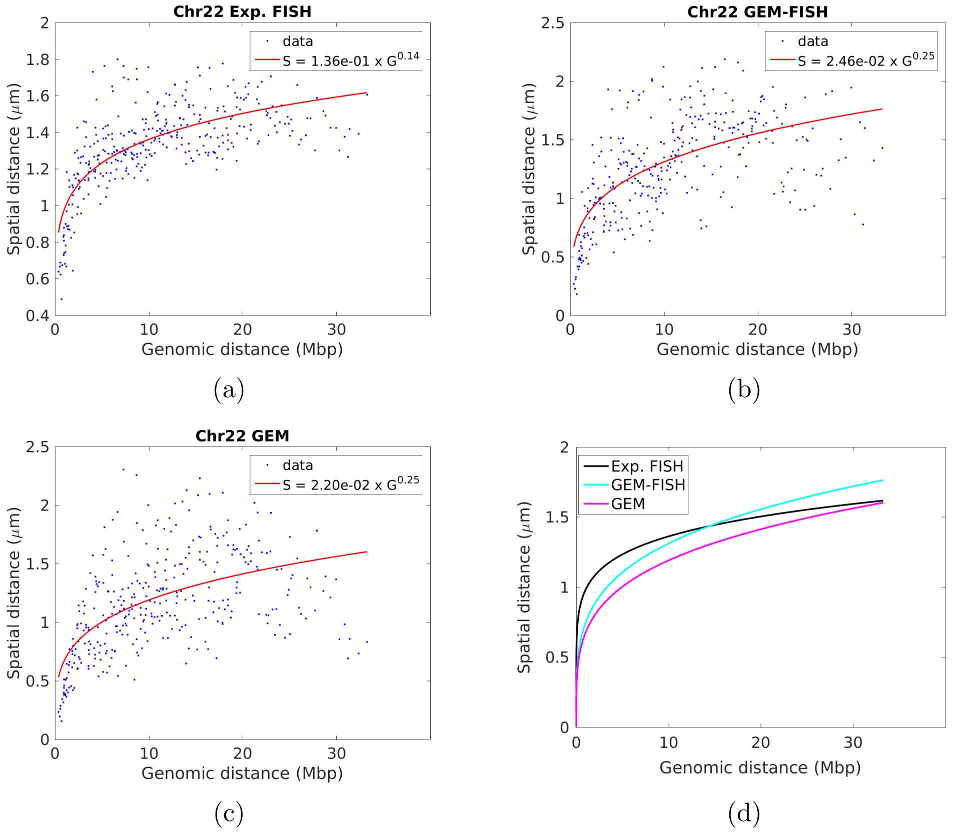

Supplementary Figure 22. The scatter plots and the corresponding fitting curves of spatial vs. genomic distances between TADs for Chr22, which were derived from the experimental FISH data (a), and the final 3D models reconstructed by GEM-FISH (b), and by GEM (c). (d) A combined figure for the three fitting curves shown in (a, b, and c).

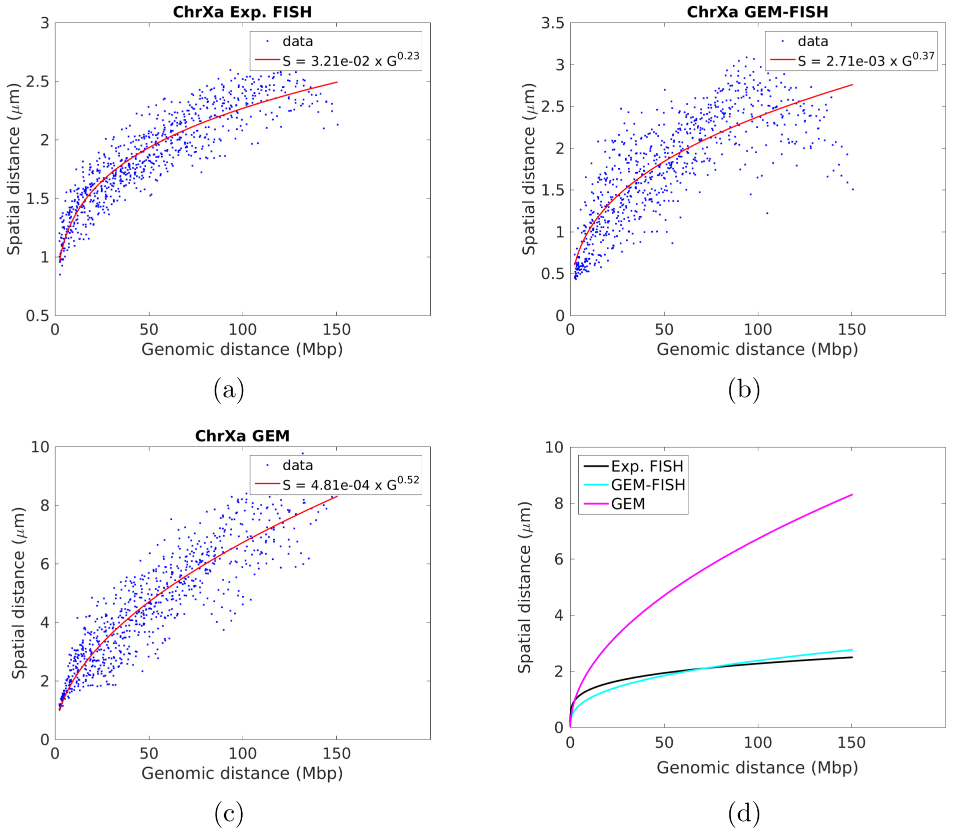

Supplementary Figure 23. The scatter plots and the corresponding fitting curves of spatial vs. genomic distances between TADs for ChrXa, which were derived from the experimental FISH data (a), and the final 3D models reconstructed by GEM-FISH (b), and by GEM (c). (d) A combined figure for the three fitting curves shown in (a, b, and c).

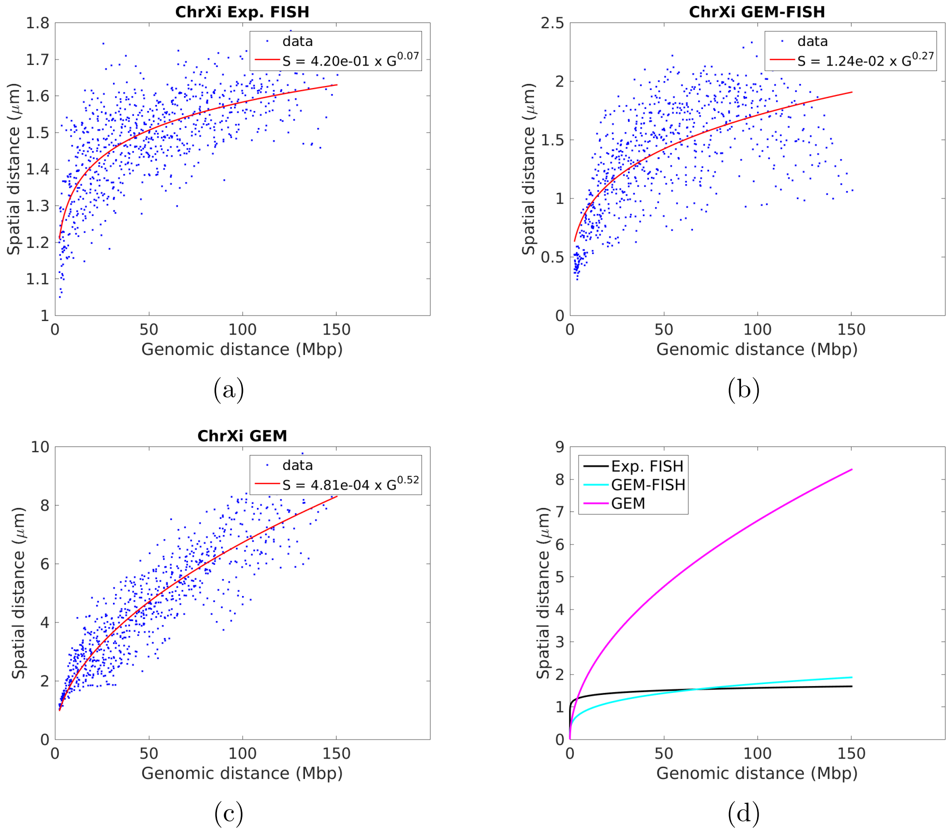

Supplementary Figure 24. The scatter plots and the corresponding fitting curves of spatial vs. genomic distances between TADs for ChrXi, which were derived from the experimental FISH data (a), and the final 3D models reconstructed by GEM-FISH (b), and by GEM (c). (d) A combined figure for the three fitting curves shown in (a, b, and c).

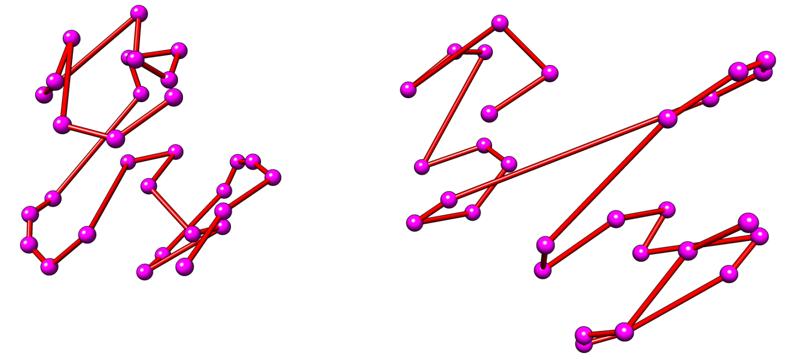

(a)

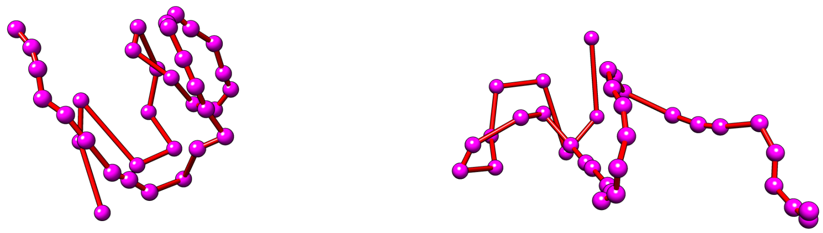

(b)

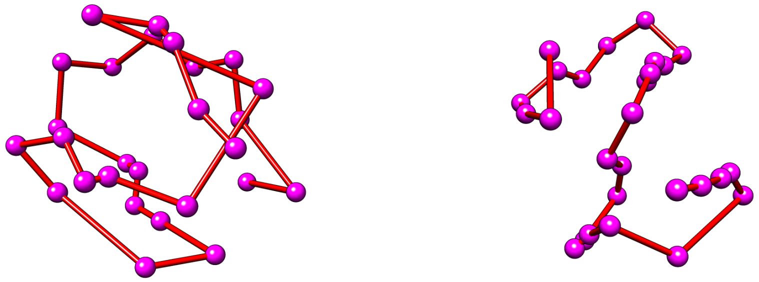

(c)

Supplementary Figure 25. The 3D models reconstructed by GEM-FISH tended to be more spherical than those reconstructed by GEM. (a, b, and c) The TAD-level resolution models derived by GEM-FISH (left) and GEM (right) for Chrs 20, 21, and 22, respectively.

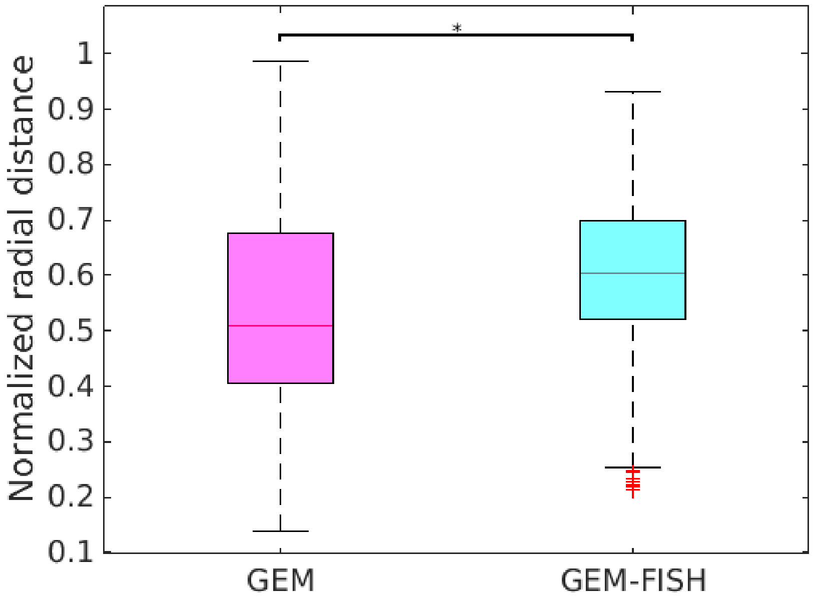

Supplementary Figure 26. The expressed genes tend to lie closer to the chromosome surface in the 3D models reconstructed by GEM-FISH than in those reconstructed by GEM. $N = 1091$ points in the final 3D chromosome models, corresponding to the 5 Kbp genomic loci of the expressed genes in Chrs 20, 21, and 22 for both GEM and GEM-FISH. *: p-value $< 10^{-20}$, Wilcoxon rank sum test. The expressed genes were defined as those with expression values > 20 (as in [19]). For the boxplots, the top and bottom lines of each box represent the 75th and 25th percentiles of the samples, respectively. The line inside each box represents the median of the samples. The upper and lower lines above and below the boxes are the whiskers. Red points marked by '+' represent outliers, which represent the observations beyond 1.5 times interquartile range away from the top or the bottom of the box.

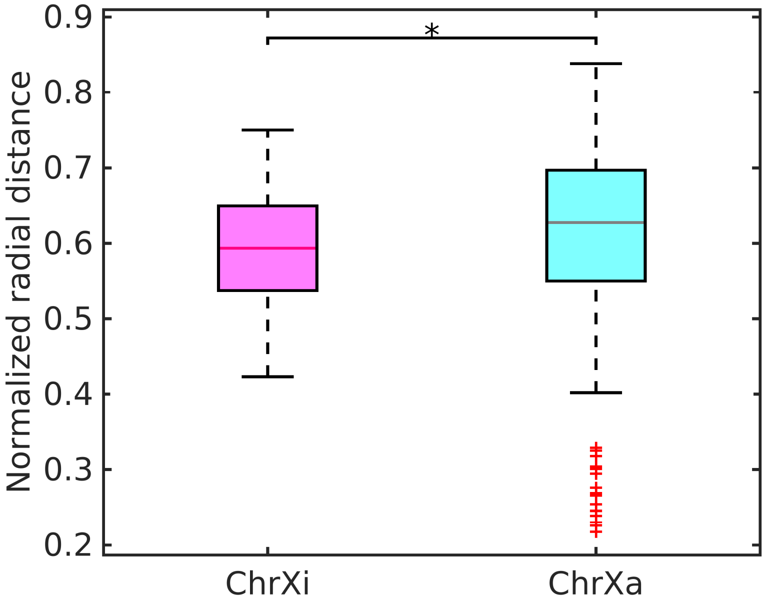

Supplementary Figure 27. According to the final 3D models reconstructed by GEM-FISH, the expressed genes of ChrX tend to lie closer to the chromosome surface in ChrXa (in which the genes are active) than in ChrXi (in which the genes are inactive, except for those that escape the X-inactivation). $N = 216$ points in the final 3D chromosome models of both ChrXi and ChrXa, corresponding to the 5 Kbp genomic loci of the expressed genes in ChrX. *: p-value < 0.002, one-tailed Wilcoxon rank sum test. For the boxplots, the top and bottom lines of each box represent the 75th and 25th percentiles of the samples, respectively. The line inside each box represents the median of the samples. The upper and lower lines above and below the boxes are the whiskers. Red points marked by '+' represent outliers, which represent the observations beyond 1.5 times interquartile range away from the top or the bottom of the box.

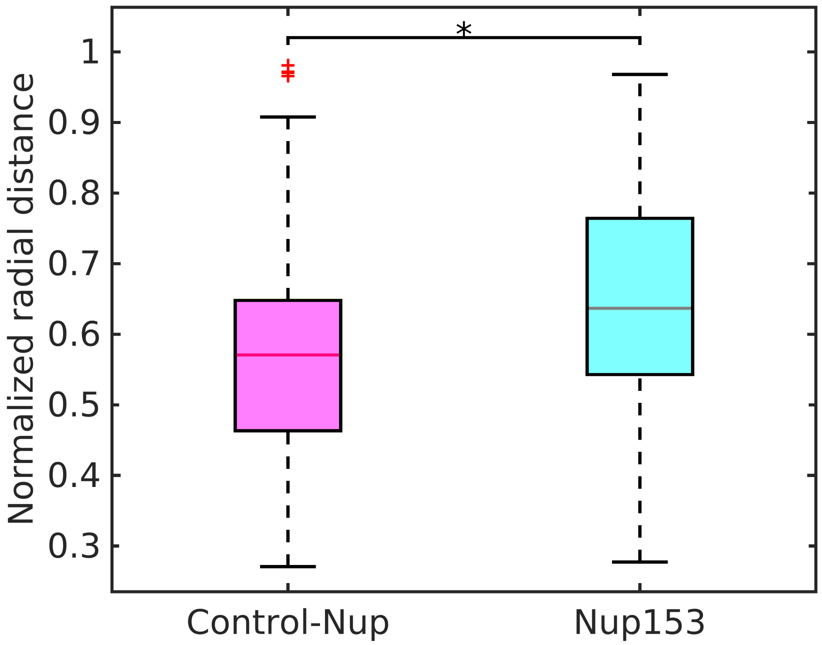

Supplementary Figure 28. The genomic sites that are known to interact with the NPC component Nup153 tend to lie closer to the chromosome surface in the 3D models reconstructed by GEM-FISH than the randomly selected loci of the same genomic lengths (denoted by Control-Nup). $N = 502$ points in the final 3D chromosome models, corresponding to the 5 Kbp genomic loci, for both Control-Nup and Nup153 regions. *: p-value $< 10^{-8}$, Wilcoxon rank sum test. For the boxplots, the top and bottom lines of each box represent the 75th and 25th percentiles of the samples, respectively. The line inside each box represents the median of the samples. The upper and lower lines above and below the boxes are the whiskers. Red points marked by '+' represent outliers, which represent the observations beyond 1.5 times interquartile range away from the top or the bottom of the box.

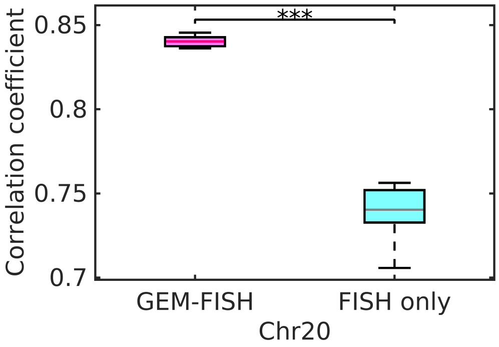
***
Correlation coefficient
0.85
0.8
0.75
0.7
GEM-FISH
FISH only
Chr20

(a)

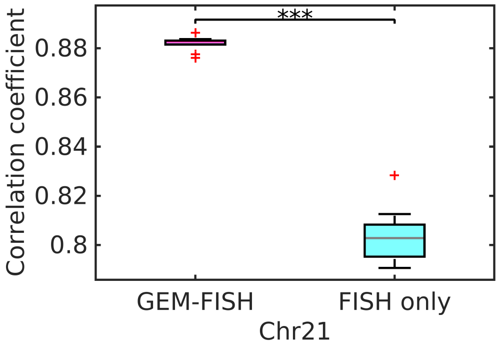
***
Correlation coefficient
0.88
0.86
0.84
0.82
0.8
GEM-FISH
FISH only
Chr21

(b)

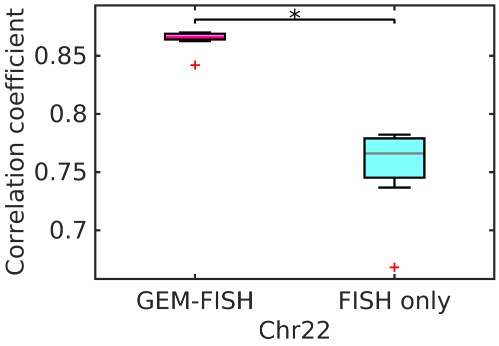
*
Correlation coefficient
0.85
0.8
0.75
0.7
GEM-FISH
FISH only
Chr22

(c)

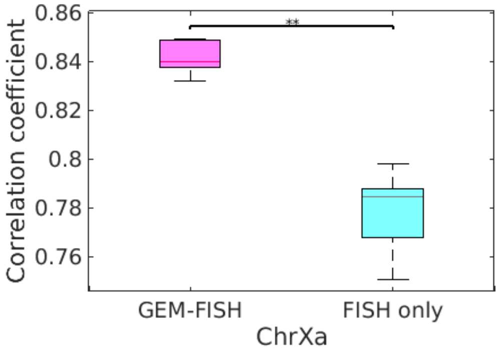
**
Correlation coefficient
0.86
0.84
0.82
0.8
0.78
0.76
GEM-FISH
FISH only
ChrXa

(d)

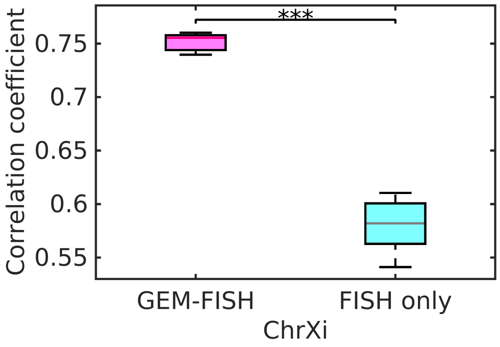
***
Correlation coefficient
0.75
0.7
0.65
0.6
0.55
GEM-FISH
FISH only
ChrXi

(e)

Supplementary Figure 29. Pearson correlation coefficients between the inverse of Hi-C contact frequencies and the corresponding spatial distances derived from the 3D models reconstructed by GEM-FISH and a modified version of GEM-FISH that used only FISH data for different chromosomes. $N = 10$ independent 3D models reconstructed by both GEM-FISH and its modified version that uses only FISH data for the five chromosomes. ***: p-value $< 10^{-13}$, **: p-value $< 10^{-9}$, *: p-value $< 10^{-7}$, Wilcoxon rank sum test. For the boxplots, the top and bottom lines of each box represent the 75th and 25th percentiles of the samples, respectively. The line inside each box represents the median of the samples. The upper and lower lines above and below the boxes are the whiskers. Red points marked by '+' represent outliers, which represent the observations beyond 1.5 times interquartile range away from the top or the bottom of the box.

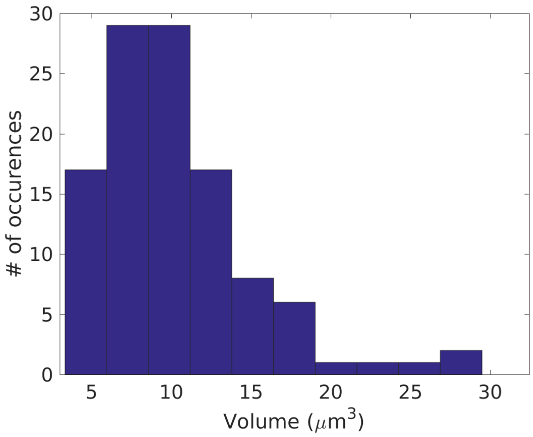
30
25
20
15
10
5
0
# of occurences
5
10
15
20
25
30
Volume (μm^3)

(a)

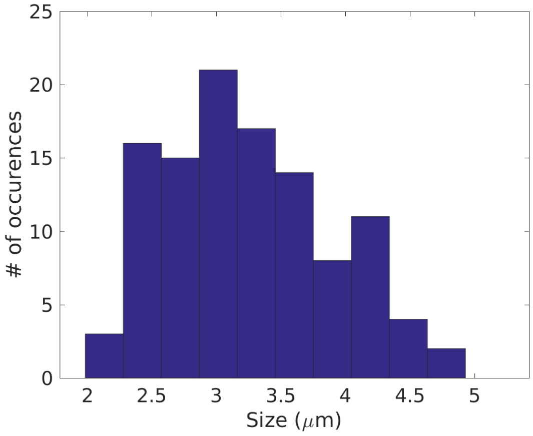
25
20
15
10
5
0
# of occurences
2
2.5
3
3.5
4
4.5
5
Size (μm)

(b)

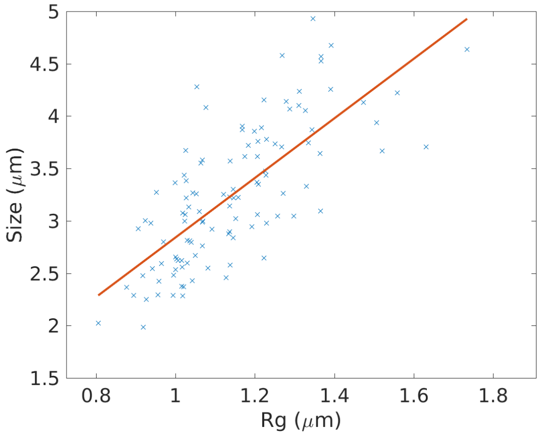
5
4.5
4
3.5
3
2.5
2
1.5
Size (μm)
0.8
1
1.2
1.4
1.6
1.8
Rg (μm)

(c)

Supplementary Figure 30. Distributions of the volumes and sizes of Chr20, and the relation between its size and its radius of gyration obtained from experimental FISH data. (a) Histogram showing the distribution of the volumes of Chr20. (b) Histogram showing the distribution of the sizes of Chr20. (c) The approximate linear relation between the radius of gyration and the size of Chr20. Data from 111 cells [2] were used to generate (a), (b), and (c).

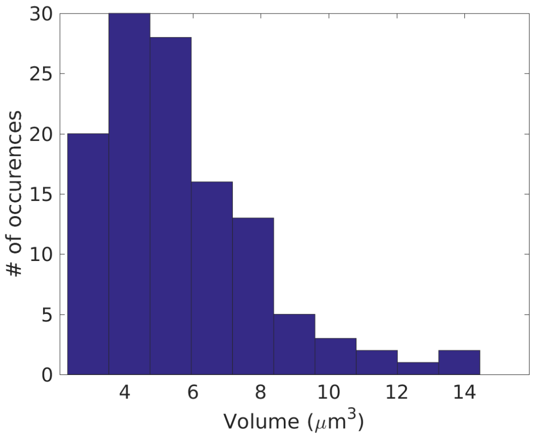

# of occurences
30
25
20
15
10
5
0
4
6
8
10
12
14
Volume (μm³)

(a)

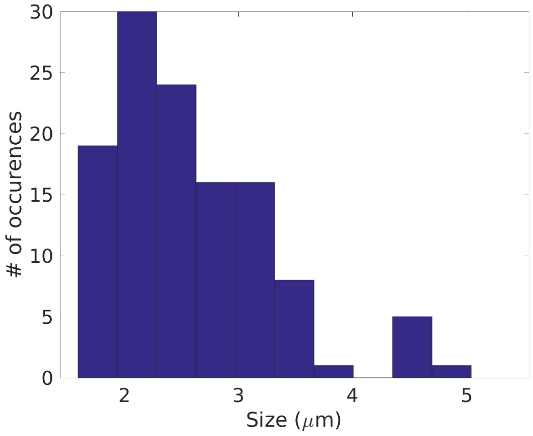

# of occurences
30
25
20
15
10
5
0
2
3
4
5
Size (μm)

(b)

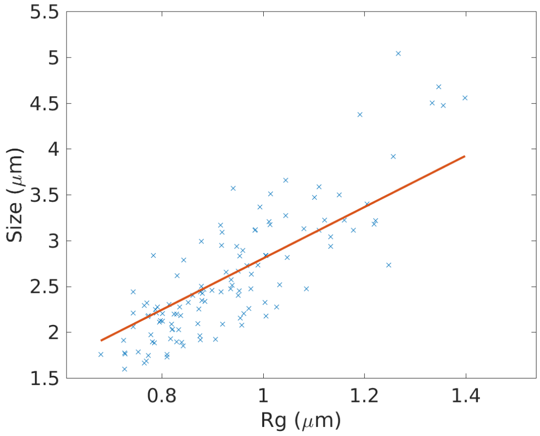

Size (μm)
5.5
5
4.5
4
3.5
3
2.5
2
1.5
0.8
1
1.2
1.4
Rg (μm)

(c)

Supplementary Figure 31. Distributions of the volumes and sizes of Chr21, and the relation between its size and its radius of gyration obtained from experimental FISH data. (a) Histogram showing the distribution of the volumes of Chr21. (b) Histogram showing the distribution of the sizes of Chr21. (c) The approximate linear relation between the radius of gyration and the size of Chr21. Data from 120 cells [2] were used to generate (a), (b), and (c).

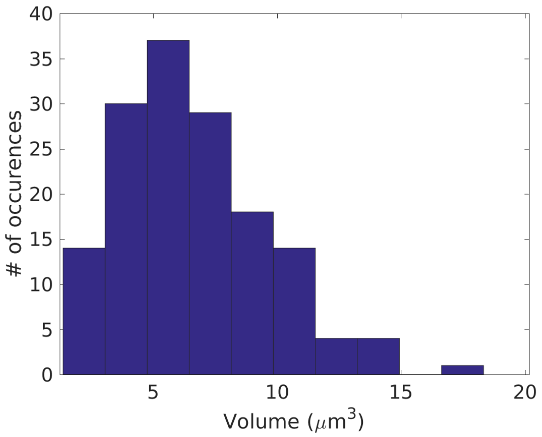

(a)

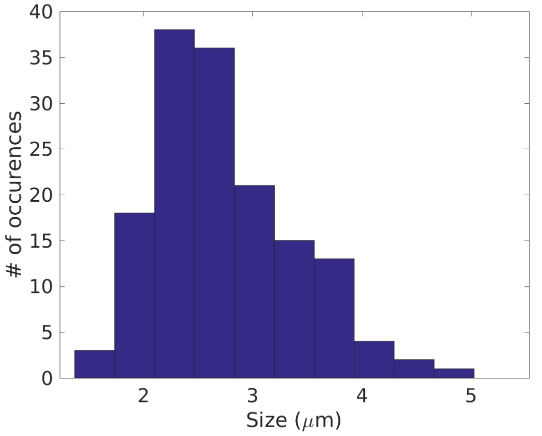

(b)

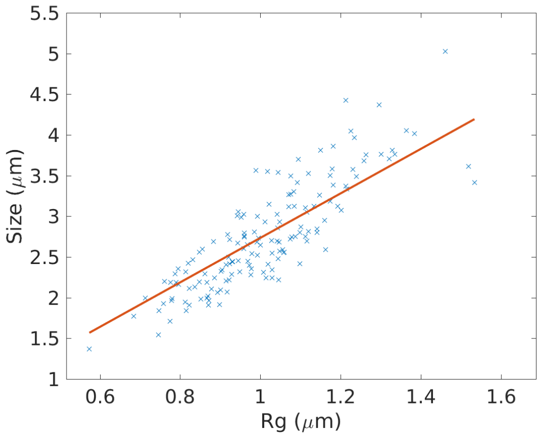

(c)

Supplementary Figure 32. Distributions of the volumes and sizes of Chr22, and the relation between its size and its radius of gyration obtained from experimental FISH data. (a) Histogram showing the distribution of the volumes of Chr22. (b) Histogram showing the distribution of the sizes of Chr22. (c) The approximate linear relation between the radius of gyration and the size of Chr22. Data from 151 cells [2] were used to generate (a), (b), and (c).

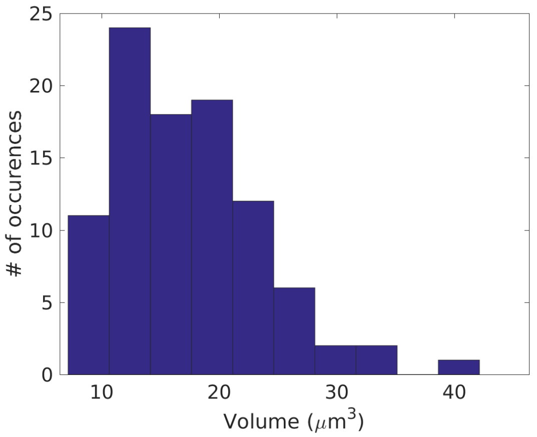

# of occurences
Volume (μm^3)

(a)

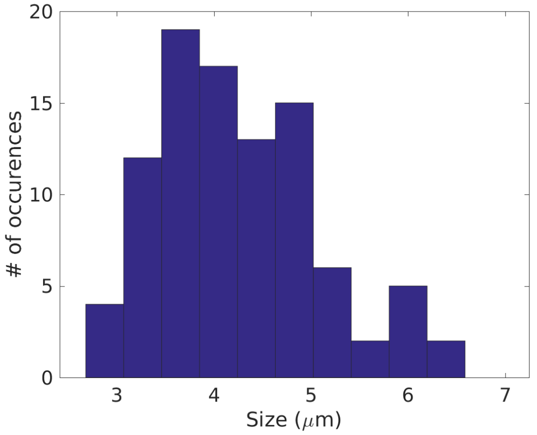

# of occurences
Size (μm)

(b)

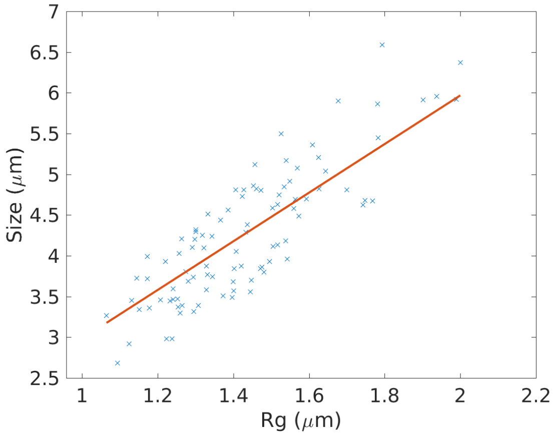

Size (μm)
Rg (μm)

(c)

Supplementary Figure 33. Distributions of the volumes and sizes of ChrXa, and the relation between its size and its radius of gyration obtained from experimental FISH data. (a) Histogram showing the distribution of the volumes of ChrXa. (b) Histogram showing the distribution of the sizes of ChrXa. (c) The approximate linear relation between the radius of gyration and the size of ChrXa. Data from 95 cells [2] were used to generate (a), (b), and (c).

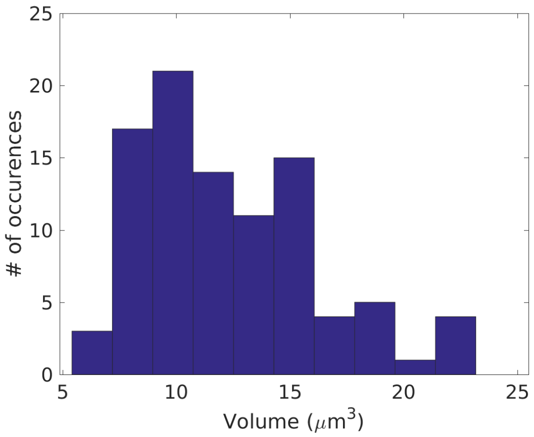

(a)

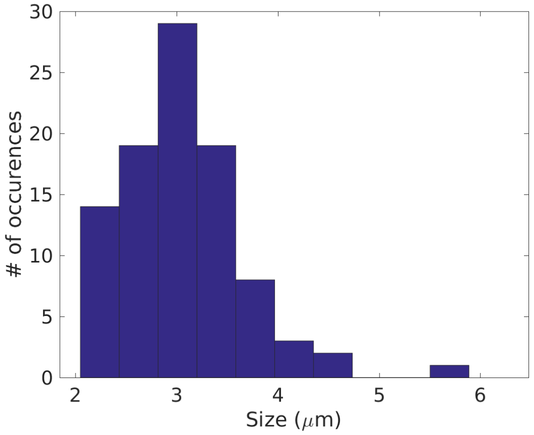

(b)

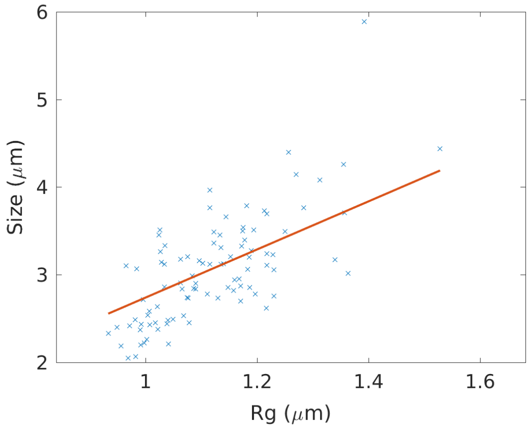

(c)

Supplementary Figure 34. Distributions of the volumes and sizes of ChrXi, and the relation between its size and its radius of gyration obtained from experimental FISH data. (a) Histogram showing the distribution of the volumes of ChrXi. (b) Histogram showing the distribution of the sizes of ChrXi. (c) The approximate linear relation between the radius of gyration and the size of ChrXi. Data from 95 cells [2] were used to generate (a), (b), and (c).

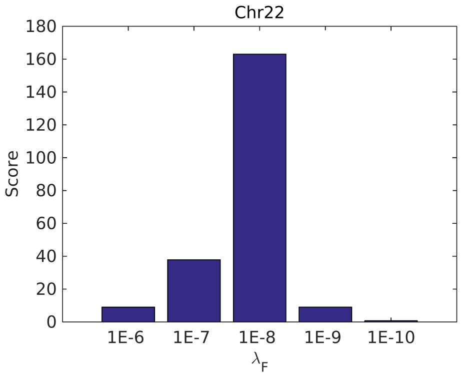

(a)

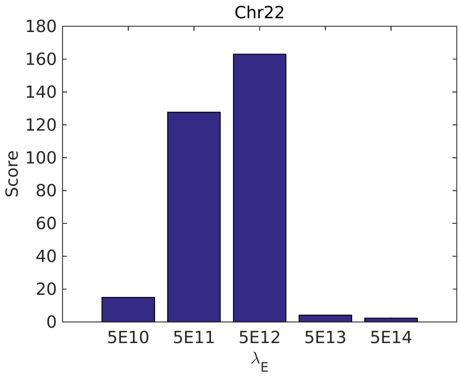

(b)

Supplementary Figure 35. The values of the scoring function for Chr22 with different parameter settings. (a) The values of the scoring function when fixing the value of $\lambda_E = 5 \times 10^{12}$ and changing the value of λ_F. (b) The values of the scoring function when fixing the value of $\lambda_F = 1 \times 10^{-8}$ and changing the value of λ_E.

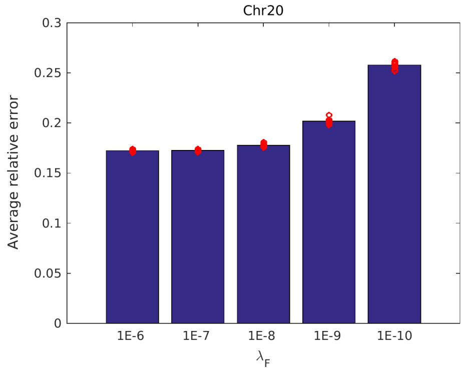
Chr20
Average relative error
0.3
0.25
0.2
0.15
0.1
0.05
0
1E-6
1E-7
1E-8
1E-9
1E-10
λ_F

(a)

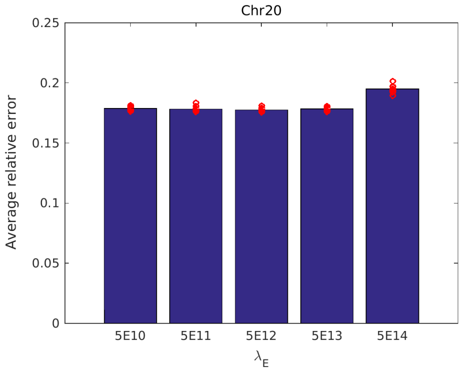
Chr20
Average relative error
0.25
0.2
0.15
0.1
0.05
0
5E10
5E11
5E12
5E13
5E14
λ_E

(b)

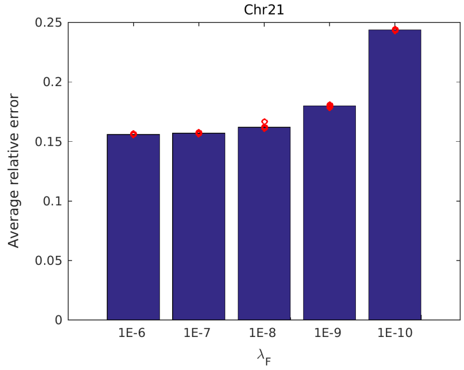
Chr21
Average relative error
0.25
0.2
0.15
0.1
0.05
0
1E-6
1E-7
1E-8
1E-9
1E-10
λ_F

(c)

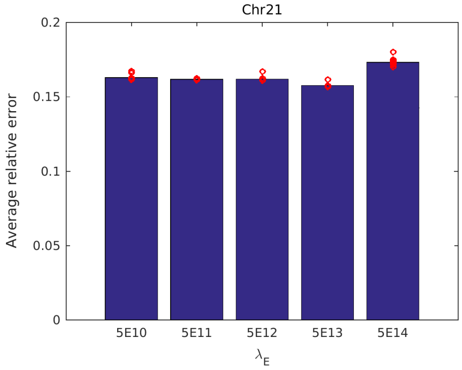
Chr21
Average relative error
0.2
0.15
0.1
0.05
0
5E10
5E11
5E12
5E13
5E14
λ_E

(d)

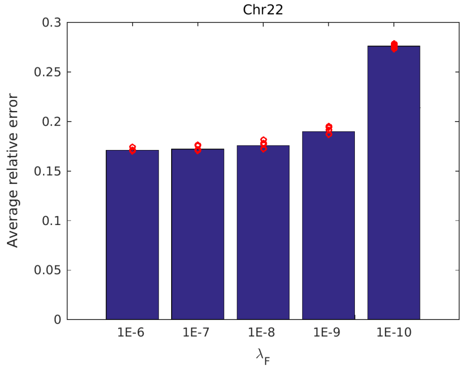
Chr22
Average relative error
0.3
0.25
0.2
0.15
0.1
0.05
0
1E-6
1E-7
1E-8
1E-9
1E-10
λ_F

(e)

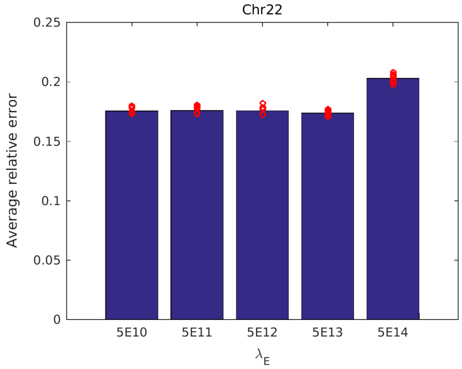
Chr22
Average relative error
0.25
0.2
0.15
0.1
0.05
0
5E10
5E11
5E12
5E13
5E14
λ_E

(f)

Supplementary Figure 36. The mean values of average relative errors when running GEM-FISH ten times with different parameter settings for Chrs 20, 21, and 22. (a, c, and e) The mean values of average relative errors when fixing the value of $\lambda_E = 5 \times 10^{12}$ and changing the value of λ_F for Chrs 20 (a), 21 (c), and 22 (e). (b, d, and f) The mean values of average relative errors when fixing the value of $\lambda_F = 1 \times 10^{-8}$ and changing the value of λ_E for Chrs 20 (b), 21 (d), and 22 (f). Source data are provided as a Source Data file.

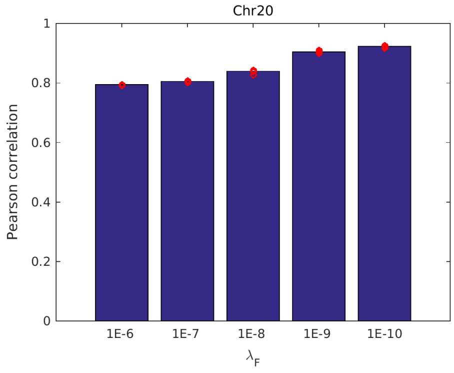
Chr20
Pearson correlation
0
0.2
0.4
0.6
0.8
1
1E-6
1E-7
1E-8
1E-9
1E-10
λ_F

(a)

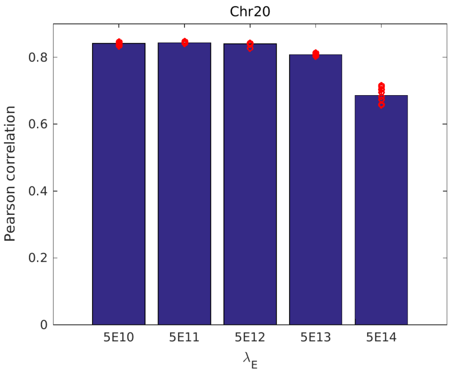
Chr20
Pearson correlation
0
0.2
0.4
0.6
0.8
5E10
5E11
5E12
5E13
5E14
λ_E

(b)

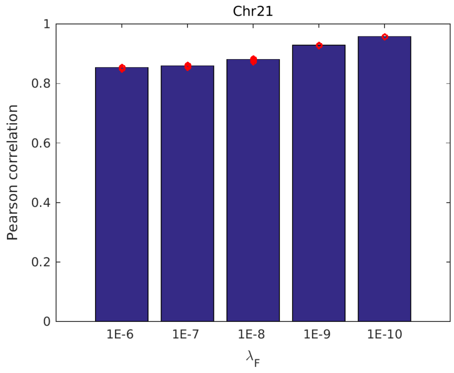
Chr21
Pearson correlation
0
0.2
0.4
0.6
0.8
1
1E-6
1E-7
1E-8
1E-9
1E-10
λ_F

(c)

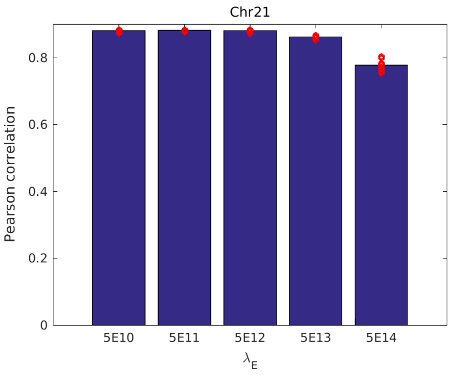
Chr21
Pearson correlation
0
0.2
0.4
0.6
0.8
5E10
5E11
5E12
5E13
5E14
λ_E

(d)

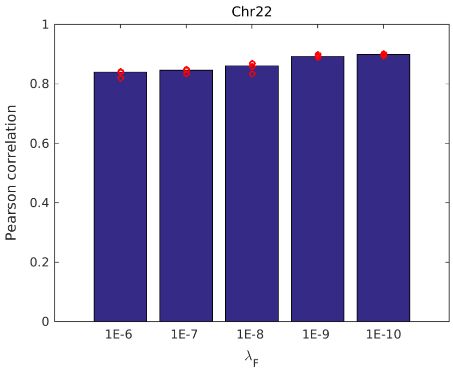
Chr22
Pearson correlation
0
0.2
0.4
0.6
0.8
1
1E-6
1E-7
1E-8
1E-9
1E-10
λ_F

(e)

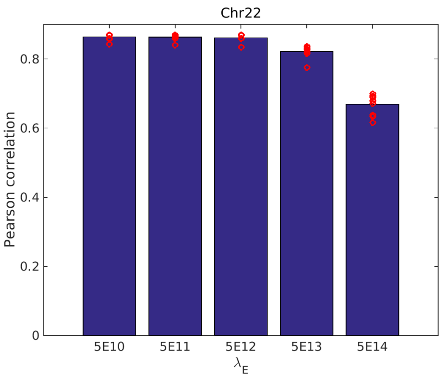
Chr22
Pearson correlation
0
0.2
0.4
0.6
0.8
5E10
5E11
5E12
5E13
5E14
λ_E

(f)

Supplementary Figure 37. The mean values of Pearson correlation coefficients between pairwise spatial distances between TADs derived from the 3D models reconstructed by GEM-FISH vs. inverse Hi-C contact frequencies when running GEM-FISH ten times with different parameter settings for Chrs 20, 21, and 22. (a, c, and e) The mean values of Pearson correlation coefficients when fixing the value of $\lambda_E = 5 \times 10^{12}$ and changing the value of λ_F for Chrs 20 (a), 21 (c), and 22 (e). (b, d, and f) The mean values of Pearson correlation coefficients when fixing the value of $\lambda_F = 1 \times 10^{-8}$ and changing the value of λ_E for Chrs 20 (b), 21 (d), and 22 (f). Source data are provided as a Source Data file.

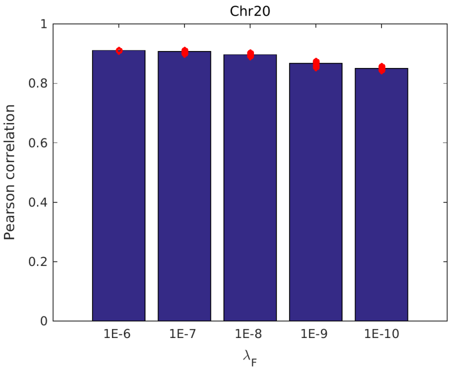
Chr20
Pearson correlation
1E-6
1E-7
1E-8
1E-9
1E-10
λ_F

(a)

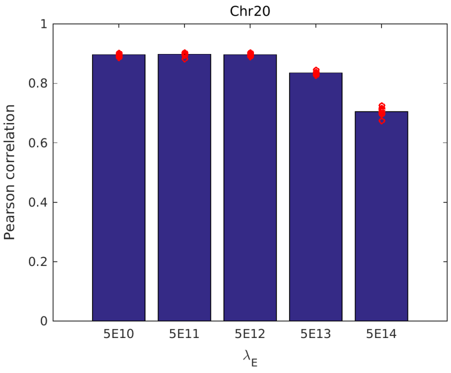
Chr20
Pearson correlation
5E10
5E11
5E12
5E13
5E14
λ_E

(b)

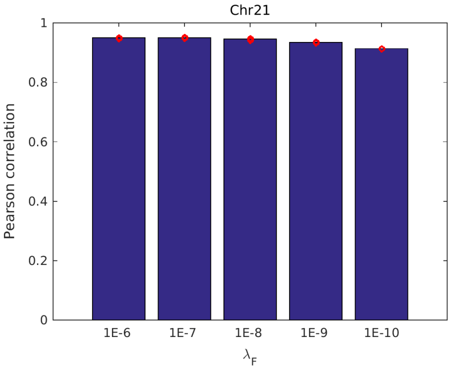
Chr21
Pearson correlation
1E-6
1E-7
1E-8
1E-9
1E-10
λ_F

(c)

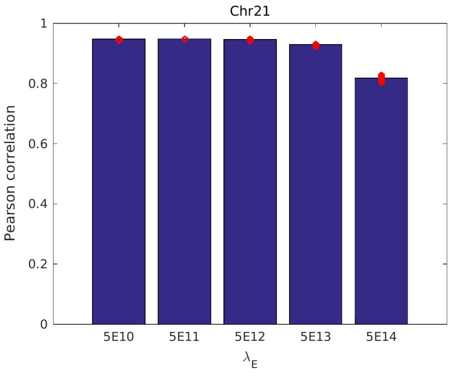
Chr21
Pearson correlation
5E10
5E11
5E12
5E13
5E14
λ_E

(d)

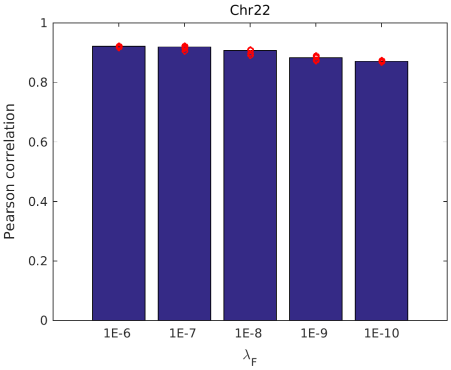
Chr22
Pearson correlation
1E-6
1E-7
1E-8
1E-9
1E-10
λ_F

(e)

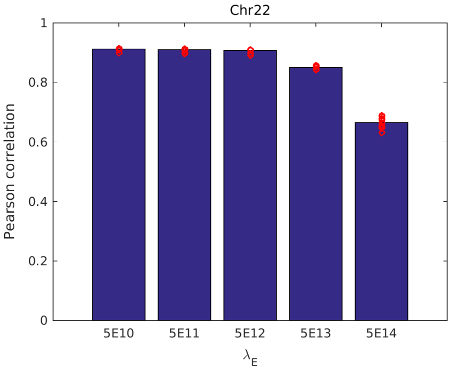
Chr22
Pearson correlation
5E10
5E11
5E12
5E13
5E14
λ_E

(f)

Supplementary Figure 38. The mean values of Pearson correlation coefficients between pairwise spatial distances between TADs derived from the 3D models reconstructed by GEM-FISH vs. experimental FISH distances when running GEM-FISH ten times with different parameter settings for Chrs 20, 21, and 22. (a, c, and e) The mean values of Pearson correlation coefficients when fixing the value of $\lambda_E = 5 \times 10^{12}$ and changing the value of λ_F for Chrs 20 (a), 21 (c), and 22 (e). (b, d, and f) The mean values of Pearson correlation coefficients when fixing the value of $\lambda_F = 1 \times 10^{-8}$ and changing the value of λ_E for Chrs 20 (b), 21 (d), and 22 (f). Source data are provided as a Source Data file.

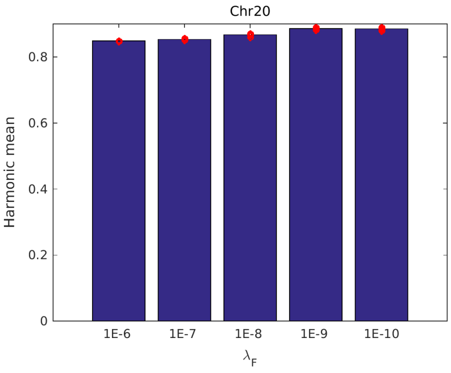
Chr20
Harmonic mean
0
0.2
0.4
0.6
0.8
1E-6
1E-7
1E-8
1E-9
1E-10
λ_F

(a)

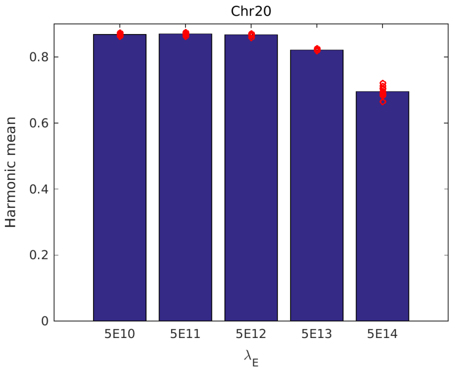
Chr20
Harmonic mean
0
0.2
0.4
0.6
0.8
5E10
5E11
5E12
5E13
5E14
λ_E

(b)

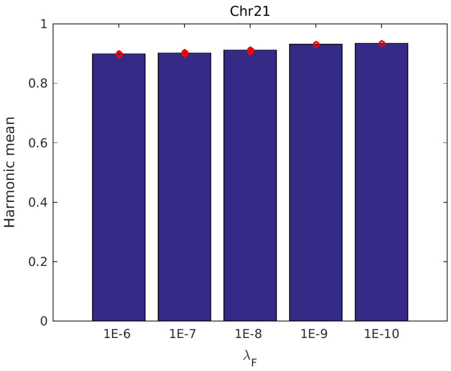
Chr21
Harmonic mean
0
0.2
0.4
0.6
0.8
1
1E-6
1E-7
1E-8
1E-9
1E-10
λ_F

(c)

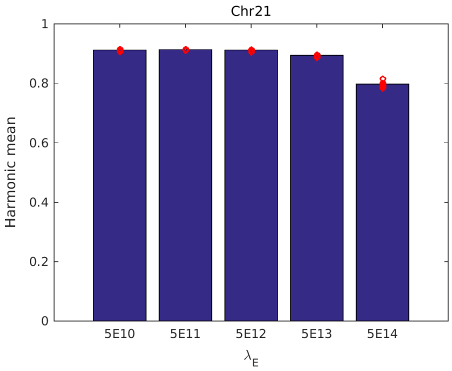
Chr21
Harmonic mean
0
0.2
0.4
0.6
0.8
1
5E10
5E11
5E12
5E13
5E14
λ_E

(d)

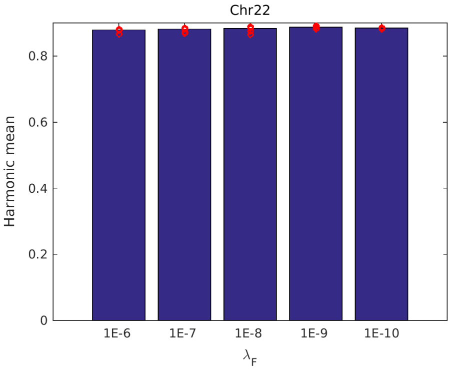
Chr22
Harmonic mean
0
0.2
0.4
0.6
0.8
1E-6
1E-7
1E-8
1E-9
1E-10
λ_F

(e)

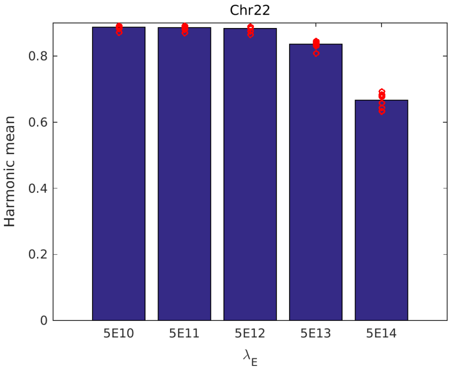
Chr22
Harmonic mean
0
0.2
0.4
0.6
0.8
5E10
5E11
5E12
5E13
5E14
λ_E

(f)

Supplementary Figure 39. The mean values of the harmonic means of Pearson correlation coefficients with both FISH distances and inverse Hi-C contact frequencies when running GEM-FISH ten times with different parameter settings for Chrs 20, 21, and 22. (a, c, and e) The mean values of the harmonic means when fixing the value of $\lambda_E = 5 \times 10^{12}$ and changing the value of λ_F for Chrs 20 (a), 21 (c), and 22 (e). (b, d, and f) The mean values of the harmonic means when fixing the value of $\lambda_F = 1 \times 10^{-8}$ and changing the value of λ_E for Chrs 20 (b), 21 (d), and 22 (f). Source data are provided as a Source Data file.

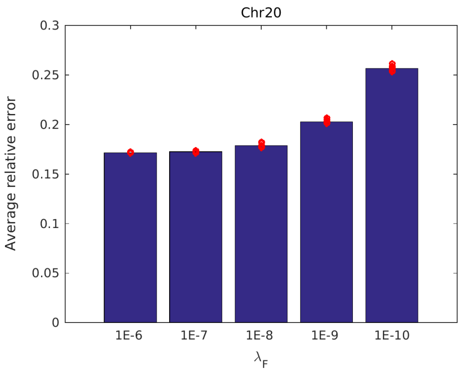
Chr20
Average relative error
0.3
0.25
0.2
0.15
0.1
0.05
0
1E-6
1E-7
1E-8
1E-9
1E-10
λ_F

(a)

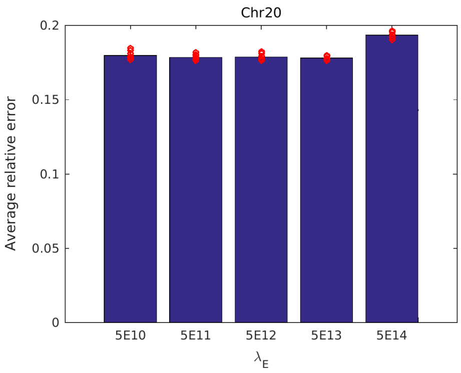
Chr20
Average relative error
0.2
0.15
0.1
0.05
0
5E10
5E11
5E12
5E13
5E14
λ_E

(b)

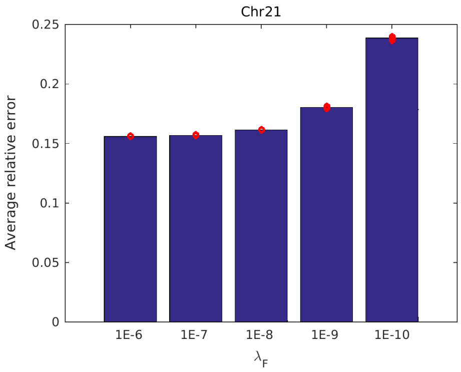
Chr21
Average relative error
0.25
0.2
0.15
0.1
0.05
0
1E-6
1E-7
1E-8
1E-9
1E-10
λ_F

(c)

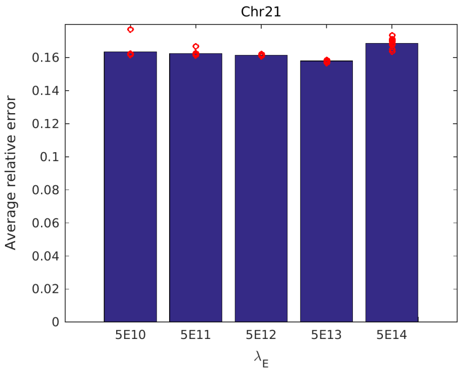
Chr21
Average relative error
0.16
0.14
0.12
0.1
0.08
0.06
0.04
0.02
0
5E10
5E11
5E12
5E13
5E14
λ_E

(d)

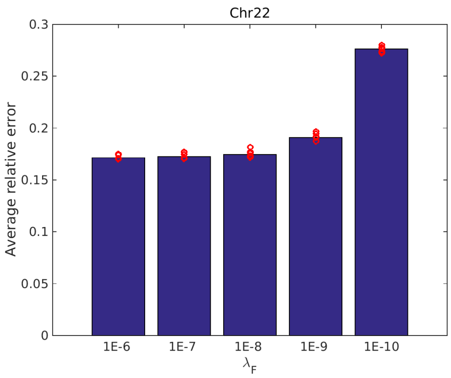
Chr22
Average relative error
0.3
0.25
0.2
0.15
0.1
0.05
0
1E-6
1E-7
1E-8
1E-9
1E-10
λ_F

(e)

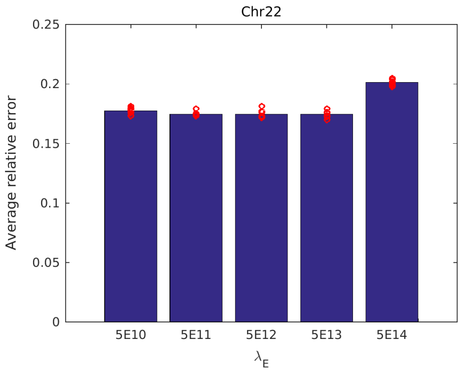
Chr22
Average relative error
0.25
0.2
0.15
0.1
0.05
0
5E10
5E11
5E12
5E13
5E14
λ_E

(f)

Supplementary Figure 40. The mean values of average relative errors when running GEM-FISH ten times with different parameter settings for Chrs 20, 21, and 22 using Hi-C data from [17] with resolution 40 Kbp. (a, c, and e) The mean values of average relative errors when fixing the value of $\lambda_E = 5 \times 10^{12}$ and changing the value of λ_F for Chrs 20 (a), 21 (c), and 22 (e). (b, d, and f) The mean values of average relative errors when fixing the value of $\lambda_F = 1 \times 10^{-8}$ and changing the value of λ_E for Chrs 20 (b), 21 (d), and 22 (f). Source data are provided as a Source Data file.

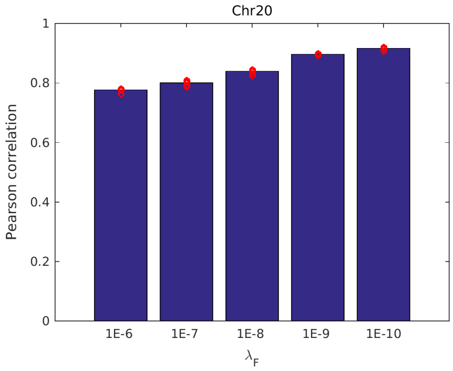
Chr20
Pearson correlation
1E-6
1E-7
1E-8
1E-9
1E-10
λ_F

(a)

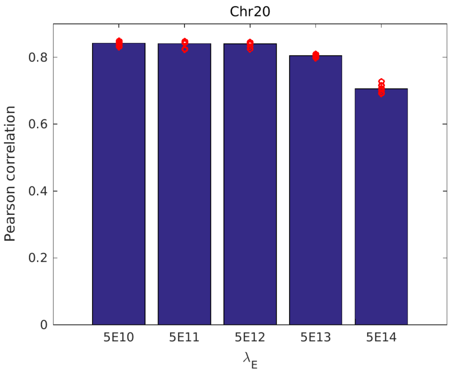
Chr20
Pearson correlation
5E10
5E11
5E12
5E13
5E14
λ_E

(b)

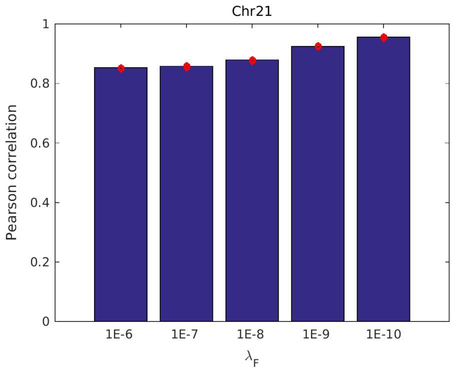
Chr21
Pearson correlation
1E-6
1E-7
1E-8
1E-9
1E-10
λ_F

(c)

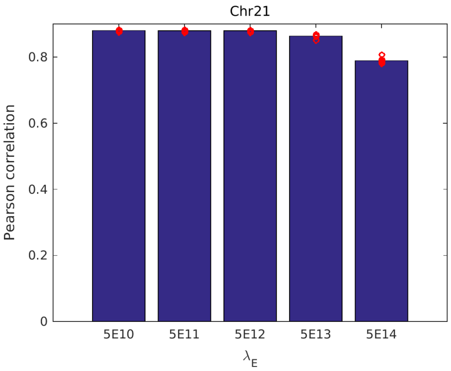
Chr21
Pearson correlation
5E10
5E11
5E12
5E13
5E14
λ_E

(d)

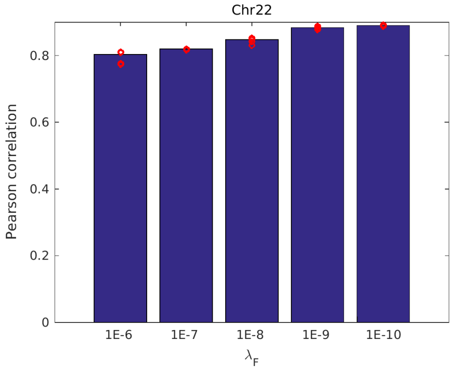
Chr22
Pearson correlation
1E-6
1E-7
1E-8
1E-9
1E-10
λ_F

(e)

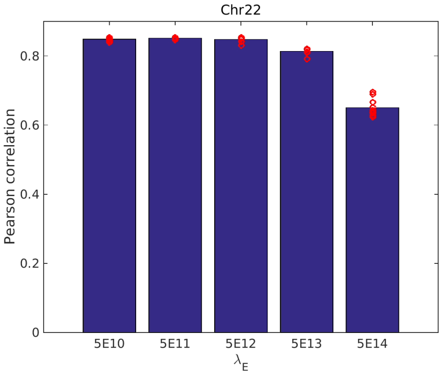
Chr22
Pearson correlation
5E10
5E11
5E12
5E13
5E14
λ_E

(f)

Supplementary Figure 41. The mean values of Pearson correlation coefficients between the pairwise spatial distances between TADs obtained from the 3D models reconstructed by GEM-FISH vs. inverse Hi-C contact frequencies when running GEM-FISH ten times with different parameter settings for Chrs 20, 21, and 22 using Hi-C data from [17] with resolution 40 Kbp. (a, c, and e) The mean values of Pearson correlation coefficients when fixing the value of $\lambda_E = 5 \times 10^{12}$ and changing the value of λ_F for Chrs 20 (a), 21 (c), and 22 (e). (b, d, and f) The mean values of Pearson correlation coefficients when fixing the value of $\lambda_F = 1 \times 10^{-8}$ and changing the value of λ_E for Chrs 20 (b), 21 (d), and 22 (f). Source data are provided as a Source Data file.

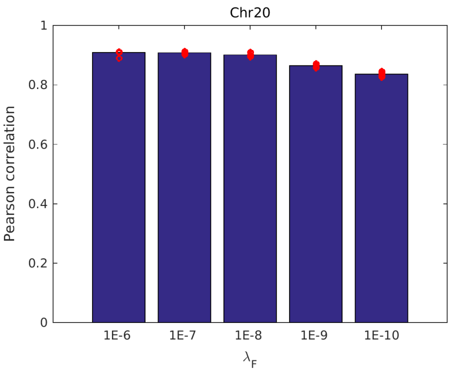
Chr20
Pearson correlation
0
0.2
0.4
0.6
0.8
1
1E-6
1E-7
1E-8
1E-9
1E-10
λF

(a)

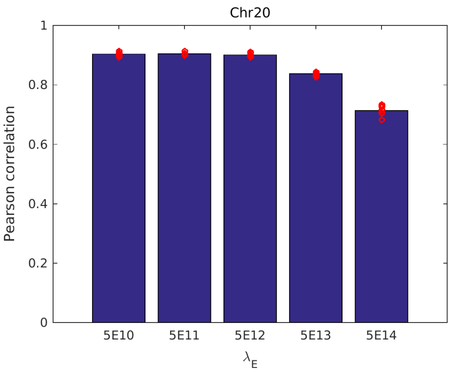
Chr20
Pearson correlation
0
0.2
0.4
0.6
0.8
1
5E10
5E11
5E12
5E13
5E14
λE

(b)

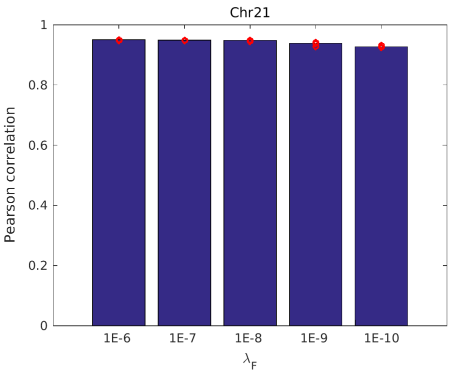
Chr21
Pearson correlation
0
0.2
0.4
0.6
0.8
1
1E-6
1E-7
1E-8
1E-9
1E-10
λF

(c)

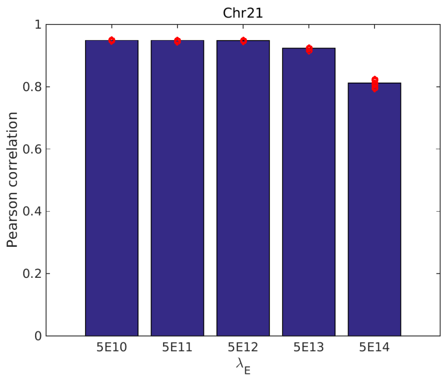
Chr21
Pearson correlation
0
0.2
0.4
0.6
0.8
1
5E10
5E11
5E12
5E13
5E14
λE

(d)

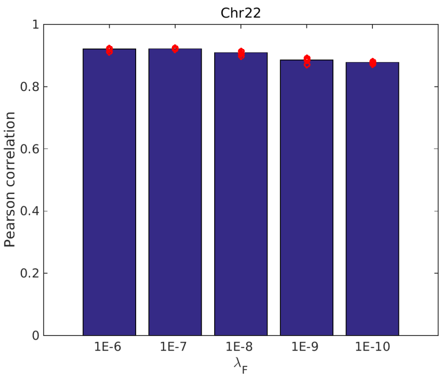
Chr22
Pearson correlation
0
0.2
0.4
0.6
0.8
1
1E-6
1E-7
1E-8
1E-9
1E-10
λF

(e)

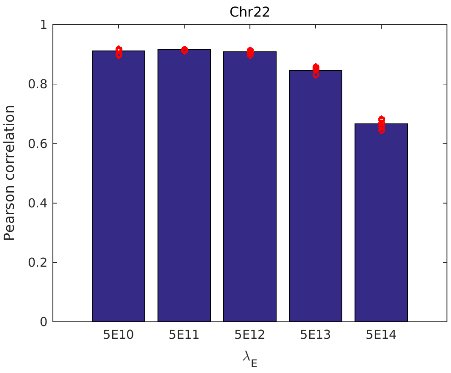
Chr22
Pearson correlation
0
0.2
0.4
0.6
0.8
1
5E10
5E11
5E12
5E13
5E14
λE

(f)

Supplementary Figure 42. The mean values of Pearson correlation coefficients between the pairwise spatial distances between TADs obtained from the 3D models reconstructed by GEM-FISH vs. experimental FISH distances when running GEM-FISH ten times with different parameter settings for Chrs 20, 21, and 22 using Hi-C data from [17] with resolution 40 Kbp. (a, c, and e) The mean values of Pearson correlation coefficients when fixing the value of $\lambda_E = 5 \times 10^{12}$ and changing the value of λ_F for Chrs 20 (a), 21 (c), and 22 (e). (b, d, and f) The mean values of Pearson correlation coefficients when fixing the value of $\lambda_F = 1 \times 10^{-8}$ and changing the value of λ_E for Chrs 20 (b), 21 (d), and 22 (f). Source data are provided as a Source Data file.

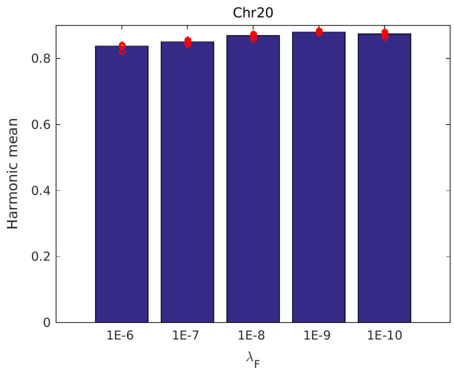
Chr20
Harmonic mean
0
0.2
0.4
0.6
0.8
1E-6
1E-7
1E-8
1E-9
1E-10
λ_F

(a)

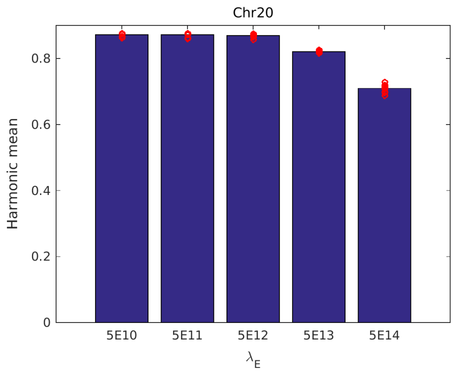
Chr20
Harmonic mean
0
0.2
0.4
0.6
0.8
5E10
5E11
5E12
5E13
5E14
λ_E

(b)

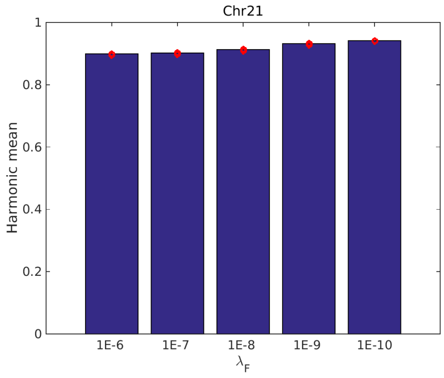
Chr21
Harmonic mean
0
0.2
0.4
0.6
0.8
1
1E-6
1E-7
1E-8
1E-9
1E-10
λ_F

(c)

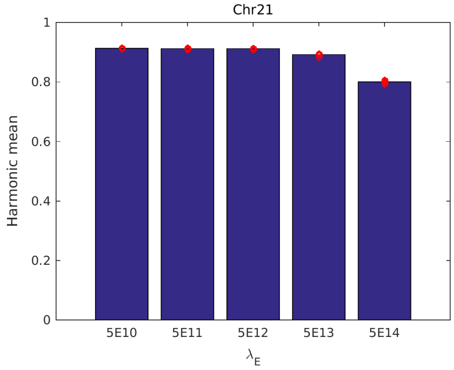
Chr21
Harmonic mean
0
0.2
0.4
0.6
0.8
1
5E10
5E11
5E12
5E13
5E14
λ_E

(d)

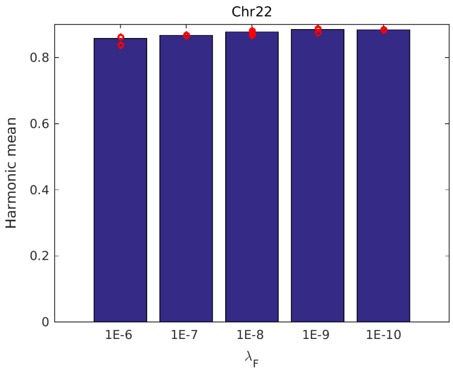
Chr22
Harmonic mean
0
0.2
0.4
0.6
0.8
1E-6
1E-7
1E-8
1E-9
1E-10
λ_F

(e)

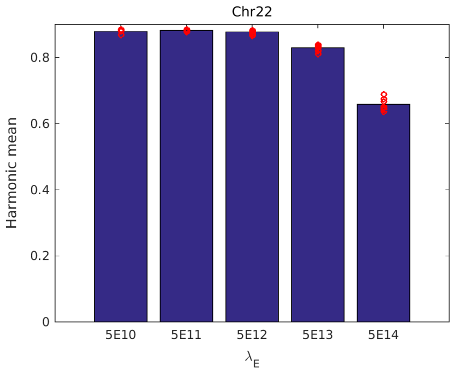
Chr22
Harmonic mean
0
0.2
0.4
0.6
0.8
5E10
5E11
5E12
5E13
5E14
λ_E

(f)

Supplementary Figure 43. The mean values of the harmonic means of Pearson correlation coefficients with both FISH distances and inverse Hi-C contact frequencies when running GEM-FISH ten times with different parameter settings for Chrs 20, 21, and 22 using Hi-C data from [17] with resolution 40 Kbp. (a, c, and e) The mean values of the harmonic means when fixing the value of $\lambda_E = 5 \times 10^{12}$ and changing the value of λ_F for Chrs 20 (a), 21 (c), and 22 (e). (b, d, and f) The mean values of the harmonic means when fixing the value of $\lambda_F = 1 \times 10^{-8}$ and changing the value of λ_E for Chrs 20 (b), 21 (d), and 22 (f). Source data are provided as a Source Data file.

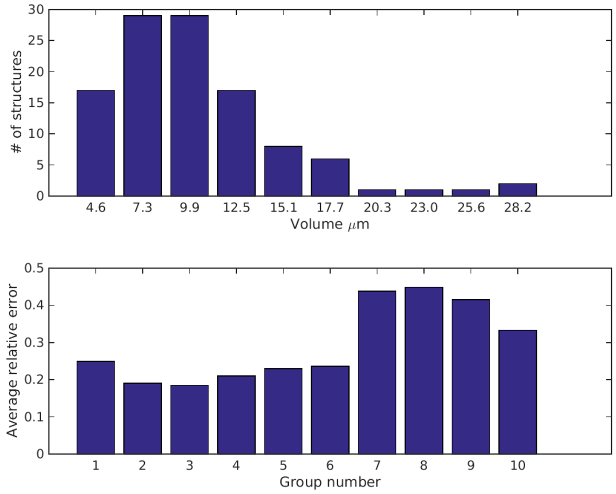

Supplementary Figure 44. The agreement between the heterogeneity of individual chromosomes measured by FISH experiments and the average modeling results derived by GEM-FISH for Chr20. Top: the histograms showing the distribution of the number of structures within individual 10 groups classified according to the volumes of the minimum bounding boxes enclosing the spatial positions of TADs measured in FISH experiments. Bottom: the average relative errors obtained by comparing the average distance matrix of each corresponding group with the average 3D model reconstructed by GEM-FISH.

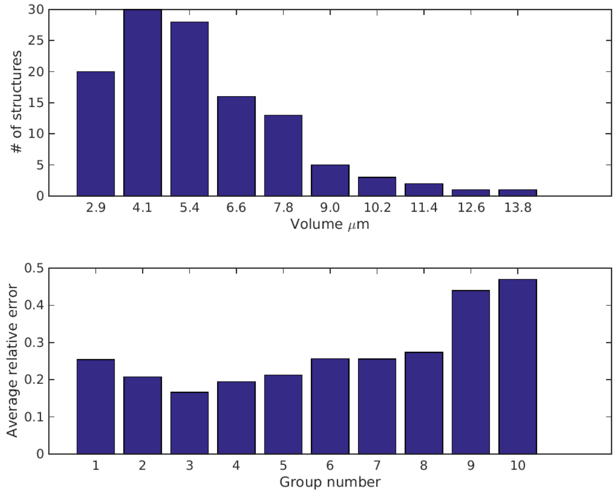

Supplementary Figure 45. The agreement between the heterogeneity of individual chromosomes measured by FISH experiments and the average modeling results derived by GEM-FISH for Chr21. Top: the histograms showing the distribution of the number of structures within individual 10 groups classified according to the volumes of the minimum bounding boxes enclosing the spatial positions of TADs measured in FISH experiments. Bottom: the average relative errors obtained by comparing the average distance matrix of each corresponding group with the average 3D model reconstructed by GEM-FISH.

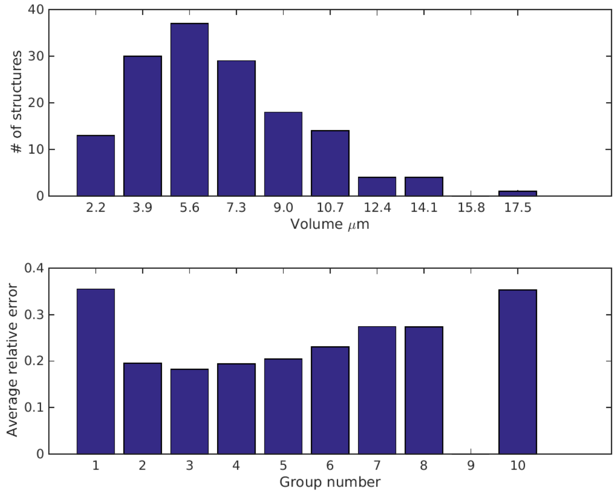

Supplementary Figure 46. The agreement between the heterogeneity of individual chromosomes measured by FISH experiments and the average modeling results derived by GEM-FISH for Chr22. Top: the histograms showing the distribution of the number of structures within individual 10 groups classified according to the volumes of the minimum bounding boxes enclosing the spatial positions of TADs measured in FISH experiments. Bottom: the average relative errors obtained by comparing the average distance matrix of each corresponding group with the average 3D model reconstructed by GEM-FISH.

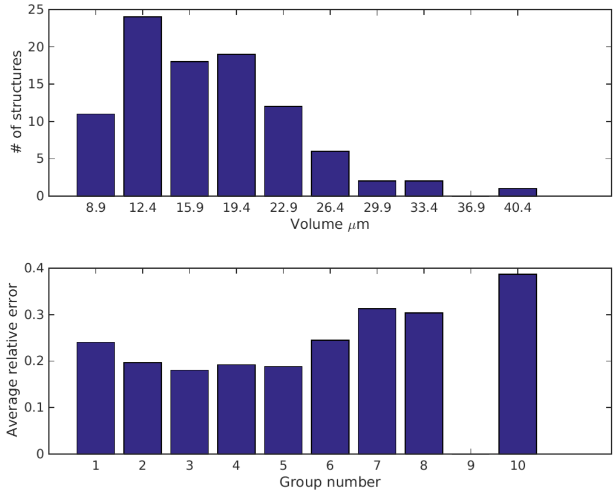

Supplementary Figure 47. The agreement between the heterogeneity of individual chromosomes measured by FISH experiments and the average modeling results derived by GEM-FISH for ChrXa. Top: the histograms showing the distribution of the number of structures within individual 10 groups classified according to the volumes of the minimum bounding boxes enclosing the spatial positions of TADs measured in FISH experiments. Bottom: the average relative errors obtained by comparing the average distance matrix of each corresponding group with the average 3D model reconstructed by GEM-FISH.

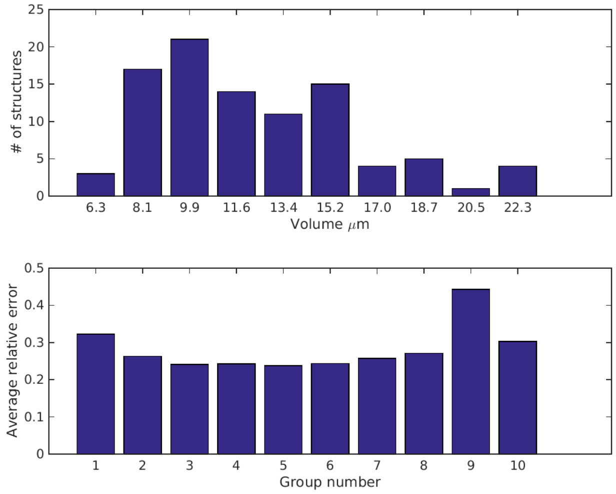

Supplementary Figure 48. The agreement between the heterogeneity of individual chromosomes measured by FISH experiments and the average modeling results derived by GEM-FISH for ChrXi. Top: the histograms showing the distribution of the number of structures within individual 10 groups classified according to the volumes of the minimum bounding boxes enclosing the spatial positions of TADs measured in FISH experiments. Bottom: the average relative errors obtained by comparing the average distance matrix of each corresponding group with the average 3D model reconstructed by GEM-FISH.

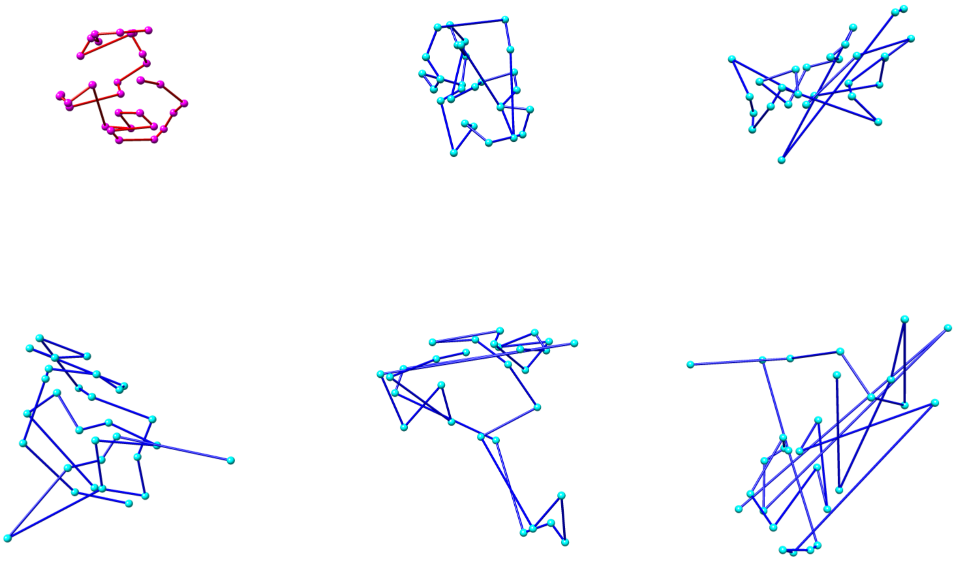

(a)

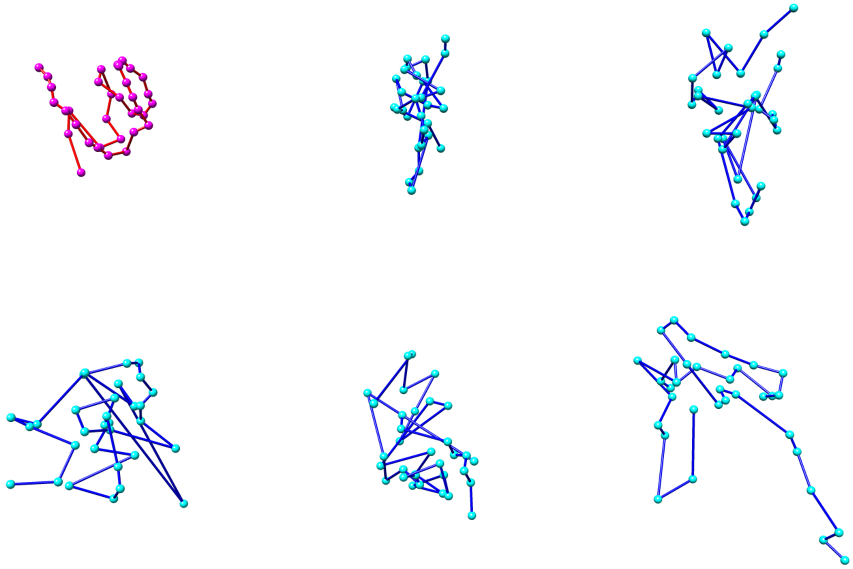

(b)

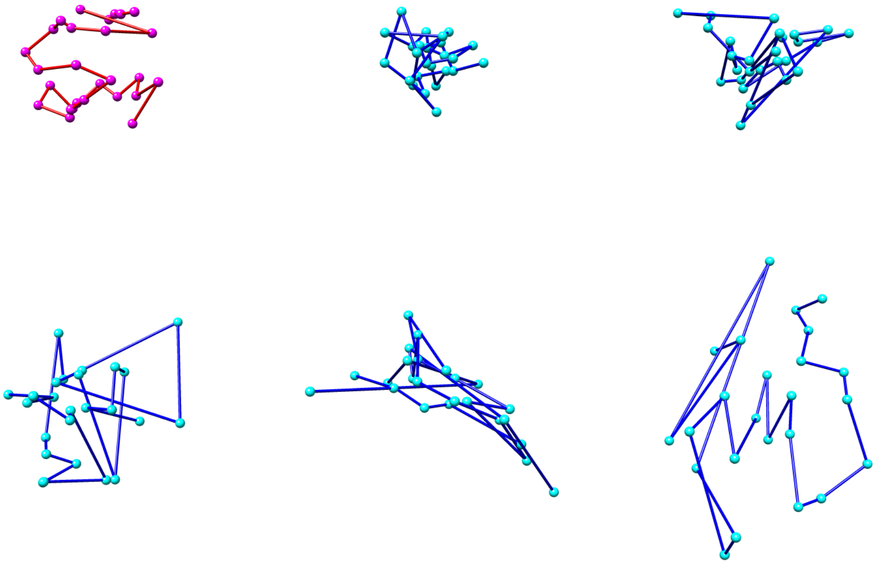

(c)

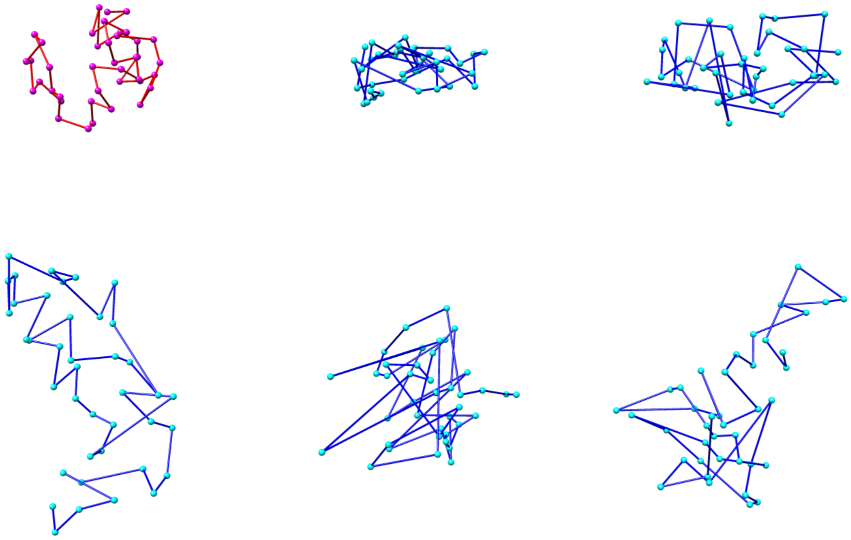

(d)

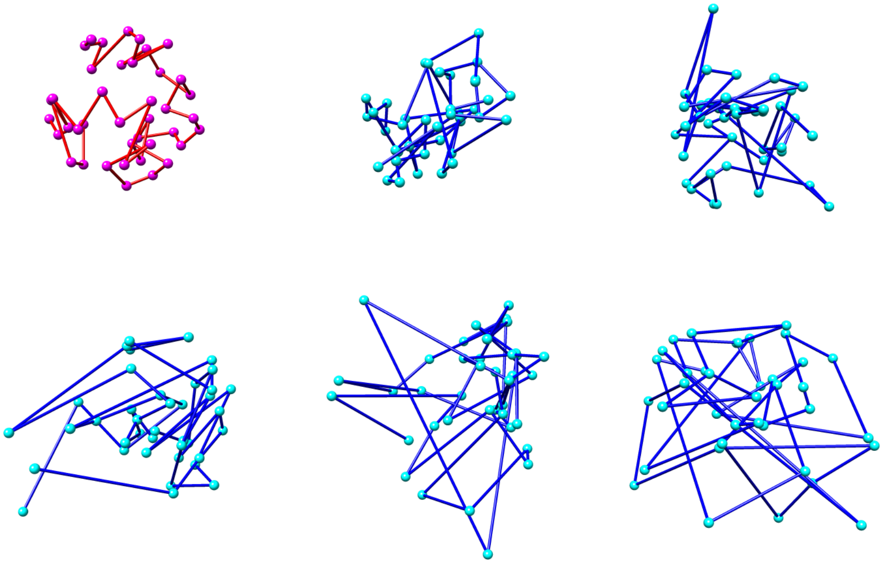

(e)

Supplementary Figure 49. FISH conformations of various copies of the same chromosome show deviation from each other and from the average 3D model reconstructed by GEM-FISH. (a-e) Plots of average 3D model (in magenta) and FISH conformations of five randomly picked copies (in cyan) of Chr20 (a), Chr21 (b), Chr22 (c), ChrXa (d), and Chr(Xi).

Supplementary References

1. Zhu, G. *et al.* Reconstructing spatial organizations of chromosomes through manifold learning. *Nucleic Acids Res.* gky065 (2018).

2. Wang, S. *et al.* Spatial organization of chromatin domains and compartments in single chromosomes. *Science* **353**, 598–602 (2016).

3. Cremer, T. & Cremer, M. Chromosome territories. *Cold Spring Harb. Perspect. Biol.* **2** (2010).

4. Rudnick, J. & Gaspari, G. The shapes of random walks. *Science* **237**, 384–389 (1987).

5. Stevens, T. J. *et al.* 3D structures of individual mammalian genomes studied by single-cell Hi-C. *Nature* **544**, 59–64 (2017).

6. Williams, R. R. Transcription and the territory: the ins and outs of gene positioning. *Trends Genet.* **19**, 298 – 302 (2003).

7. Chaumeil, J., Le Baccon, P., Wutz, A. & Heard, E. A novel role for xist rna in the formation of a repressive nuclear compartment into which genes are recruited when silenced. *Genes Dev.* **20**, 2223–2237 (2006).

8. Ibarra, A., Benner, C., Tyagi, S., Cool, J. & Hetzer, M. W. Nucleoporin-mediated regulation of cell identity genes. *Genes Dev.* **30**, 2253–2258 (2016).

9. Rabut, G., Doye, V. & Ellenberg, J. Mapping the dynamic organization of the nuclear pore complex inside single living cells. *Nat. Cell Biol.* **6**, 1114–1121 (2004).

10. Rao, S. *et al.* A 3D map of the human genome at kilobase resolution reveals principles of chromatin looping. *Cell* **159**, 1665–1680 (2014).

11. Di Pierro, M., Cheng, R. R., Lieberman Aiden, E., Wolynes, P. G. & Onuchic, J. N. De novo prediction of human chromosome structures: Epigenetic marking patterns encode genome architecture. *Proc. Natl. Acad. Sci. U.S.A.* **114**, 12126–12131 (2017).

12. Boettiger, A. N. *et al.* Super-resolution imaging reveals distinct chromatin folding for different epigenetic states. *Nature* **529**, 418–422 (2016).

13. Lizio, M. *et al.* Gateways to the fantom5 promoter level mammalian expression atlas. *Genome Biol.* **16**, 22 (2015).

14. `http://fantom.gsc.riken.jp/5/datafiles/latest/extra/Enhancers/`.

15. Fudenberg, G. & Imakaev, M. Fish-ing for captured contacts: towards reconciling fish and 3c. *Nat. Methods* **14**, 673–679 (2017).

16. Varoquaux, N., Ay, F., Noble, W. S. & Vert, J.-P. A statistical approach for inferring the 3D structure of the genome. *Bioinformatics* **30**, i26–i33 (2014).

17. Dixon, J. R. *et al.* Topological domains in mammalian genomes identified by analysis of chromatin interactions. *Nature* **485**, 376–380 (2012).

18. Pettersen, E. F. *et al.* UCSF Chimera - A visualization system for exploratory research and analysis. *J Comput Chem* **25**, 1605–1612 (2004).

19. Di Pierro, M., Zhang, B., Aiden, E. L., Wolynes, P. G. & Onuchic, J. N. Transferable model for chromosome architecture. *Proc. Natl. Acad. Sci. U.S.A.* **113**, 12168–12173 (2016).
